# What proportion of people have long-term pain after total hip or knee replacement? An update of a systematic review and meta-analysis

**DOI:** 10.1136/bmjopen-2024-088975

**Published:** 2025-05-21

**Authors:** Hung-Yuan Cheng, Andrew David Beswick, Wendy Bertram, Mohammad Ammar Siddiqui, Rachael Gooberman-Hill, Michael R Whitehouse, Vikki Wylde

**Affiliations:** 1 Bristol Medical School, University of Bristol, Bristol, UK; 2 NIHR Bristol Biomedical Research Centre, University Hospitals Bristol and Weston NHS Foundation Trust, Bristol, UK

**Keywords:** Chronic Pain, Systematic Review, Meta-Analysis, Hip, Knee, ORTHOPAEDIC & TRAUMA SURGERY

## Abstract

**Abstract:**

**Objectives:**

To update our previous systematic review to synthesise latest data on the prevalence of long-term pain in patients who underwent total hip replacement (THR) or total knee replacement (TKR). We aim to describe the prevalence estimates and trends in this review.

**Design:**

Systematic review and meta-analysis.

**Data sources:**

Update searches were conducted in MEDLINE and Embase databases from 1 January 2011 to 17 February 2024. Citation tracking was used to identify additional studies.

**Eligibility criteria:**

We included prospective cohort studies reporting long-term pain after THR or TKR at 3, 6, 12 and 24 months postoperative.

**Data extraction and synthesis:**

Two reviewers independently identified studies as eligible. One reviewer conducted data extraction, checked by a second reviewer. The risk of bias assessment was performed using Hoy’s checklist. Bayesian, random-effects meta-analysis was used to synthesise the results.

**Results:**

For TKR, 68 studies with 89 time points, including 598 498 patients, were included. Multivariate meta-analysis showed a general decrease in pain proportions over time: 21.9% (95% CrI 15.6% to 29.4%) at 3 months, 14.1% (10.9% to 17.9%) at 6 months, 12.6% (9.9% to 15.9%) at 12 months and 14.6% (9.5% to 22.4%) at 24 months. Considerable heterogeneity, unrelated to examined moderators, was indicated by substantial prediction intervals in the univariate models. Substantial loss to follow-up and risk of bias led to low confidence in the results. For THR, only 11 studies were included, so it was not possible to describe the trend. Univariate meta-analysis estimated 13.8% (8.5% to 20.1%) and 13.7% (4.8% to 31.0%) of patients experiencing long-term pain 6 and 12 months after THR, respectively, though concerns in risk of bias results reduced confidence in these findings.

**Conclusions:**

Our review suggests that approximately 22% of patients report pain 3 months post-TKR, with 12%–15% experiencing long-term pain up to 2 years. At least 14% report pain 6–12 months after THR. Given the prevalence of chronic postsurgical pain, implementing existing and developing new preventive and management strategies is crucial for optimal patient outcomes.

**PROSPERO registration number:**

CRD42023475498.

**Strengths and limitations of this study**

- We updated a previous review using the latest review methodology, including Bayesian, multivariate meta-analysis and risk of bias assessment, to summarise the prevalence rates reported across studies of chronic postsurgical pain in patients undergoing total knee or hip replacement.
- We included a wide range of patient-reported measures of pain across studies, which resulted in heterogeneity.
- These prevalence rates are likely underestimated due to loss to follow-up and the high risk of bias in the included studies.
- Our sensitivity and scenario analyses offer readers plausible and robust prevalence estimates.

## Introduction

 The primary reason that people with osteoarthritis undergo joint replacement surgery is because of persistent pain that has failed to improve with non-invasive management.^1 2^ About 100 000 each of primary total knee and hip replacements were performed in the UK in 2022,^3 4^ and in Organisation for Economic Co-operation and Development countries in 2015, over 1.5 million primary knee and nearly 1.7 million primary hip replacements were performed.^5^ The number of people with osteoarthritis is projected to increase,^6 7^ and even in Germany, a country with a declining population, rates of joint replacement are predicted to rise due to the increasing use of knee replacement in younger people and the increasing number of older people requiring hip replacement.^8^

Potential improvements in pain and functionality ability are the primary reasons that patient elect to have a hip or knee replacement, and the most important contributing factors to patient satisfaction with the outcome of surgery.^9 10^ It is important to note that pain and patient satisfaction are distinct constructs,^11^ as patient satisfaction contains broader aspects of surgical outcomes beyond solely pain relief. In the literature, the terms, such as persistent pain,^1012^,^14^ unchanged pain,^15^ residual pain^16^,^18^ and worsening pain,^19 20^ are often used to describe pain that persists despite surgery providing functional improvements and high satisfaction.^11^ It is widely recognised that some people experience continuing pain in the months and years following surgery. Our previous systematic review,^21^ with searches up to 2011, brought together longitudinal studies in representative populations receiving knee or hip replacement. We found that for a majority of people, their pain outcome was favourable, but for 10%–34% of patients the long-term pain outcome could be considered ‘unfavourable’ (moderate-to-severe pain or for whom surgery had not relieved pain) after total knee replacement (TKR) and 7%–23% after total hip replacement (THR).^21^ Together with qualitative research into patients’ experiences,^22 23^ our previous review stimulated research into the prediction, prevention, management and treatment of chronic pain after knee and hip replacement.

13 years on from the publication of our previous review, our aim is to provide updated estimates of the incidence of long-term pain after total knee and hip replacement and explore factors that may influence the rates observed. Findings will support patients, clinicians and researchers as they face the challenge of preventing and treating chronic pain after total knee or hip replacement.

## Methods

We updated our previous systematic review from our team,^21^ with follow-up intervals between 3 and 24 months postoperative. We limited the follow-up to a maximum of 24 months as pain levels often plateau by this timepoint, and new-onset pain beyond this may be related to implant failure.^24^ With the more extensive data available for outcomes after TKR in this update, we planned to establish the trend of long-term pain over time up to 24 months postoperative.

The protocol was registered with PROSPERO (CRD42023475498) and this review was reported in accordance with MOOSE^25^ (online supplemental material S1) and relevant contents in Preferred Reporting Items for Systematic Reviews and Meta-Analyses^26^ guidelines and the Cochrane handbook.^27^

### Eligibility criteria

We sought prospective cohort studies including patients representative of the general population receiving total knee or hip replacement, predominantly from advanced osteoarthritis as in our previous review.^21^ Cohorts were established preoperatively or perioperatively in hospital orthopaedic departments and joint replacement centres and followed up prospectively at any defined time between 3 and 24 months. Studies specifically of unicompartmental knee replacement or hip hemiarthroplasty, revision surgery or exclusively bilateral replacements were excluded.

### Outcome

The outcome was the proportion of people with unfavourable pain in the operated joint at 3, 6, 12 and 24 months postoperative. We adopted the term ‘unfavourable pain’ from the previous review, which serves as a collective label to include the various descriptions used by study authors—such as persistent pain, worsening pain, or residual pain—rather than as an indicator of dissatisfaction.^21 28^ In each study, unfavourable pain was defined using the study authors’ definitions or through a consensus between two reviewers with extensive research experience in pain outcome measurement in total knee and hip replacement before commencement of data extraction. Most studies used a single cut-off value, often based on a prespecified postoperative Visual Analogue Scale (VAS) or Numerical Rating Scale (NRS) score. For the few studies that provided multiple cut-off values, such as Musbahi *et al*,^18^ we selected the cut-off values that the authors concluded were the best balance between sensitivity and specificity. For studies that used general tools, such as the VAS or NRS, we only included those that reported VAS or NRS scores specific to the operated joint, rather than general VAS pain scores. To calculate the proportions, we extracted the number of recruited or followed patients as denominators and the number of patients experiencing unfavourable pain as numerators. When a percentage or rate was provided, we rounded the numbers to the nearest whole number.

### Searches

We conducted new searches of MEDLINE and Embase databases from January 2011 to 17 February 2024. The search strategies for MEDLINE and Embase are included in online supplemental material S2. Web of Science was used to track citations of the original review.^21^ Excepting the search strategy, we applied no language restrictions at any stage of the review, with Google Translate used to translate sections of relevant non-English articles. We did not contact authors as we only focused on published studies. Studies reported only as abstracts were excluded.

### Study selection and data collection

Studies identified were imported into EndNote V.21 reference management software. After the removal of duplicate records, one reviewer screened out clearly off-topic studies. Titles and abstracts of potentially relevant articles were acquired and assessed independently for eligibility by two reviewers. In cases of disagreement, a third reviewer was involved. Eligible articles identified in our previous systematic review were also included.

Data from eligible studies were entered into a Microsoft Excel spreadsheet by one reviewer with checking by a second reviewer. Extracted data were country; dates of patient recruitment; setting (single or multiple surgeons, single or multiple hospitals, registry or other; inclusion and exclusion criteria; whether routine ‘fast-track’ surgery; patient characteristics (age, sex); assessment times; number of patients at baseline, number lost to follow-up (or died or with revision surgery if reported) and number followed up; and patient reported pain outcome measure.

When more than one pain outcome was reported, we extracted them in order of preference: pain dimension data from osteoarthritis or joint specific outcome scores (Western Ontario and McMasters Universities Osteoarthritis Index (WOMAC); Knee injury and Osteoarthritis Outcome Score (KOOS); Hip injury and Osteoarthritis Outcome Score; Oxford Knee Score (OKS); Oxford Hip Score and Knee Society Scores if patient generated (KSS, IKSS); Brief Pain Inventory (BPI); pain assessed in EuroQol-5 Dimensions instruments (EQ-5D or EQ-3D); joint pain after surgery, measured on a VAS or NRS; and other measures including those developed by study authors.

### Risk of bias assessment

Two independent reviewers assessed risk of bias using the non-summative checklist described by Hoy *et al*.^29^ This checklist considers 10 aspects of study conduct relating to representation and selection, non-response (>25% of lost to follow-up as high risk), data collection and instrument used, follow-up and methods used in calculation of rates. Overall risk of bias was judged to be low, moderate or high depending on whether any of the 10 aspects gave concern.

### Data synthesis approach

Our primary aim was to describe the proportion of people experiencing unfavourable pain outcomes over time. First, we summarised the characteristics of studies and inspected their clinical heterogeneity before the synthesis using tables and figures. We then meta-analysed proportions with an unfavourable pain outcome, along with accompanying 95% credible intervals (CrIs) and median between-study heterogeneity (τ^2^ at 3, 6, 12 and 24 months’ time separately when there were more than three studies. We also used prediction intervals to aid the between-study heterogeneity interpretation.^30^ We used a Bayesian framework with a random-effects model due to anticipated heterogeneity. Vague prior distributions (eg, normal with mean 0 and variance 10^5^) on model parameters were used. Posterior outcome distributions were based on at least 25 000 simulations after a burn-in of at least 1000 to ensure convergence.

To account for the multiple time follow-ups reported in certain studies, we adopted a Bayesian, hybrid, multivariate meta-analysis of multiple factors^31^ to describe the proportions across time points by borrowing information and accounting for within-study and between-study correlations.

All analyses were performed using R V.4.3.1 on RStudio 2023.06.2+561. The runjags and metafor packages were used to produce pooled estimates, forest plots, meta-regression and subgroup analyses. The metasens package was used to generate Doi plots and the LFK index.^32^ The ggplot2 package was used to produce additional figures to explore the clinical heterogeneity in the studies.

### Exploration of heterogeneity

For potential sources of heterogeneity, we used meta-regression to explore heterogeneity for continuous factors (mean age of the population, percentage of females and baseline sample sizes) where more than 10 studies were included in the meta-analysis. For categorical factors (geographical region, settings and pain outcome instruments), we conducted subgroup analyses where more than five studies were included in the meta-analysis.

### Sensitivity analysis

In sensitivity analysis, we excluded studies with specific inclusion criteria, those focused on ‘fast-track’ surgery, studies where a proportion of people underwent unicompartmental knee replacement, studies with potentially overinclusive unfavourable pain definitions, and studies with more than 20% lost to follow-up, and studies with an overall high risk of bias. Additionally, we performed worst-best scenario analyses by estimating the proportion of people lost to follow-up who experienced unfavourable pain outcomes, incrementing by tenths from 0% to 100%, to estimate their impact on the meta-analysis results.

### Reporting bias and certainty assessment

We assessed publication bias using Doi plots and the LFK index (values between −1 and +1 indicate symmetry; values outside this interval indicate asymmetry) to aid the interpretation in cases where more than 10 studies were included in the meta-analysis. We cross-checked the clinical study register and methods section in the report to evaluate non-reporting bias. The certainty of evidence assessment was not conducted because specific tools for systematic reviews of prevalence were unavailable.

### Patient and public involvement

There was no direct patient and public involvement in this systematic review; however, it benefited from being part of the National Institute for Health and Care Research-funded Support and Treatment After Replacement (STAR) programme, which aimed to improve outcomes for patients with chronic pain after knee replacement. ^33^ Patient and public involvement was integral to STAR, and we worked throughout the programme with an existing patient forum and developed a complementary group focusing exclusively on chronic pain after TKR.

## Results

**Figure 1:**
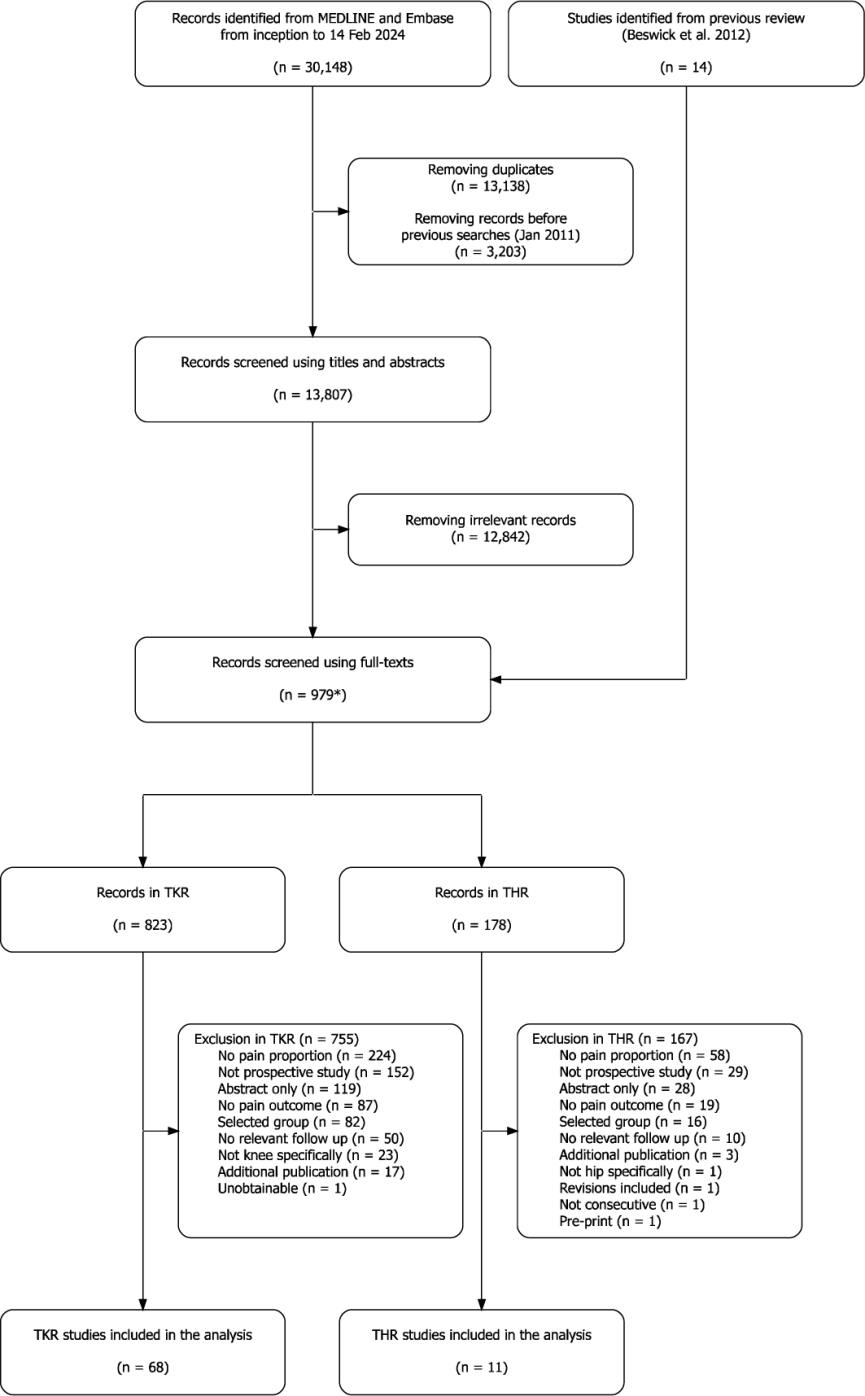
Study selection flow chart. *22 studies included both populations. THR, total hip replacement; TKR, total knee replacement.

Searches of MEDLINE, Embase, citation tracking in Web of Science and inclusion of potentially relevant articles identified in our previous review yielded a total of 13 807 records. After screening out clearly irrelevant studies by one reviewer, 979 records were screened in duplicate by two reviewers, and ultimately 68 studies with 598,498 TKR participants and 11 studies with 143 101 THR participants were included. Study selection and reasons for exclusion at the full-text stage are summarised in figure 1. Some articles from our previous review were excluded as the follow-up period was longer than 24 months.

### Total knee replacement

Individual study characteristics are summarised in online supplemental material S3. The grouped characteristics are presented in table 1. The baseline dates of data collection ranged from 1993 to 2023. Geographically, most studies were conducted in Europe (n=37) and North America (n=19). More than half of studies (n=39) collected their data at a single hospital, followed by multiple hospitals (n=18). Overall, 5 98 498 patients were included in the 68 studies with a median sample size per study of 235 (IQR 114–581). Patients in 52 studies with data had a mean age of 69.6 (SD 9.4) years, and 63% (58 to 69) were women. In terms of primary pain outcome reported, 31 studies reported multidimensional pain scales (WOMAC, OKS, KOOS, BPI or KSS/IKSS), 29 studies reported VAS or NRS pain scores and 6 studies used researchers’ own measures.

**Table 1:** Summary of TKR study characteristics

	Overall	3 months	6 months	12 months	24 months
Number of study cohorts	68	15	28	36	10
Total sample sizes	598 498	2503	550 928	36 157	13 953
Median sample size (IQR)	235(113.5–580.75)	116(95–184)	197(111.25–297)	254.5(115.5–593.75)	396.5(251.75–692.75)
Baseline time period range	1993–2023	1998–2023	1993–2023	1993–2020	1993–2019
Mean age (SD)	69.6 (9.4)(n=52*)	68.8 (9.2)(n=13*)	69.6 (9.4)(n=24*)	68.1 (9.1)(n=26*)	70 (9.3)(n=6*)
Age range	18–98(n=24)	18–90(n=7)	18–94(n=9)	25–98(n=14)	28–90(n=4)
Median % women (IQR)	63(58–69.45)	66.1(62.35–77.55)	65.55(57.65–72.475)	61.2(56.95–65.85)	63(61.03–64.75)
Primary pain outcome reported					
VAS/NRS pain	29	9	16	13	2
WOMAC pain	13	1	4	7	3
OKS pain	7	1	2	5	1
KOOS pain	6	1	1	4	1
BPI	3	1	2	2	0
KSS/IKSS pain	2	0	0	2	1
EQ-5D 5L pain/discomfort	1	0	1	0	0
Pain disturbing sleep	1	1	0	1	0
Author own question	6	1	2	2	2
Setting					
Single hospital	39	8	16	20	9
Multiple hospitals	18	0	6	12	1
Multiple surgeons	4	3	1	1	0
Single surgeon	3	3	2	2	0
National registry	2	0	1	1	0
Health region	1	0	1	0	0
Rehabilitation service	1	1	1	0	0
Country					
Australia	2	0	1	1	1
USA	17	2	8	5	4
UK	9	2	3	7	2
Spain	5	2	3	2	0
Denmark	5	1	0	5	0
France	4	1	3	2	0
Sweden	3	0	0	2	1
China	3	1	1	0	1
Belgium	2	2	1	1	0
Canada	2	0	1	1	0
Finland	2	1	0	0	1
Japan	2	1	2	1	0
Singapore	2	0	2	1	0
South Korea	2	2	0	1	0
The Netherlands	2	0	1	2	0
Hungary	1	0	0	1	0
Italy	1	0	0	1	0
New Zealand	1	0	1	1	0
Norway	1	0	0	1	0
Poland	1	0	1	0	0
Russia	1	0	0	1	0

* Only studies reported both mean and SD.

BPI, Brief Pain Inventory; EQ-5D 5L, EuroQol-5 Dimensions-5 Level; KOOS, Knee injury and Osteoarthritis Outcome Score; KSS/IKSS, Knee Society Scores if patient generated; NRS, Numerical Rating Scale; OKS, Oxford Knee Score; TKR, total knee replacement; VAS, Visual Analogue Scale; WOMAC, Western Ontario and McMasters Universities Osteoarthritis Index.

**Figure 2:**
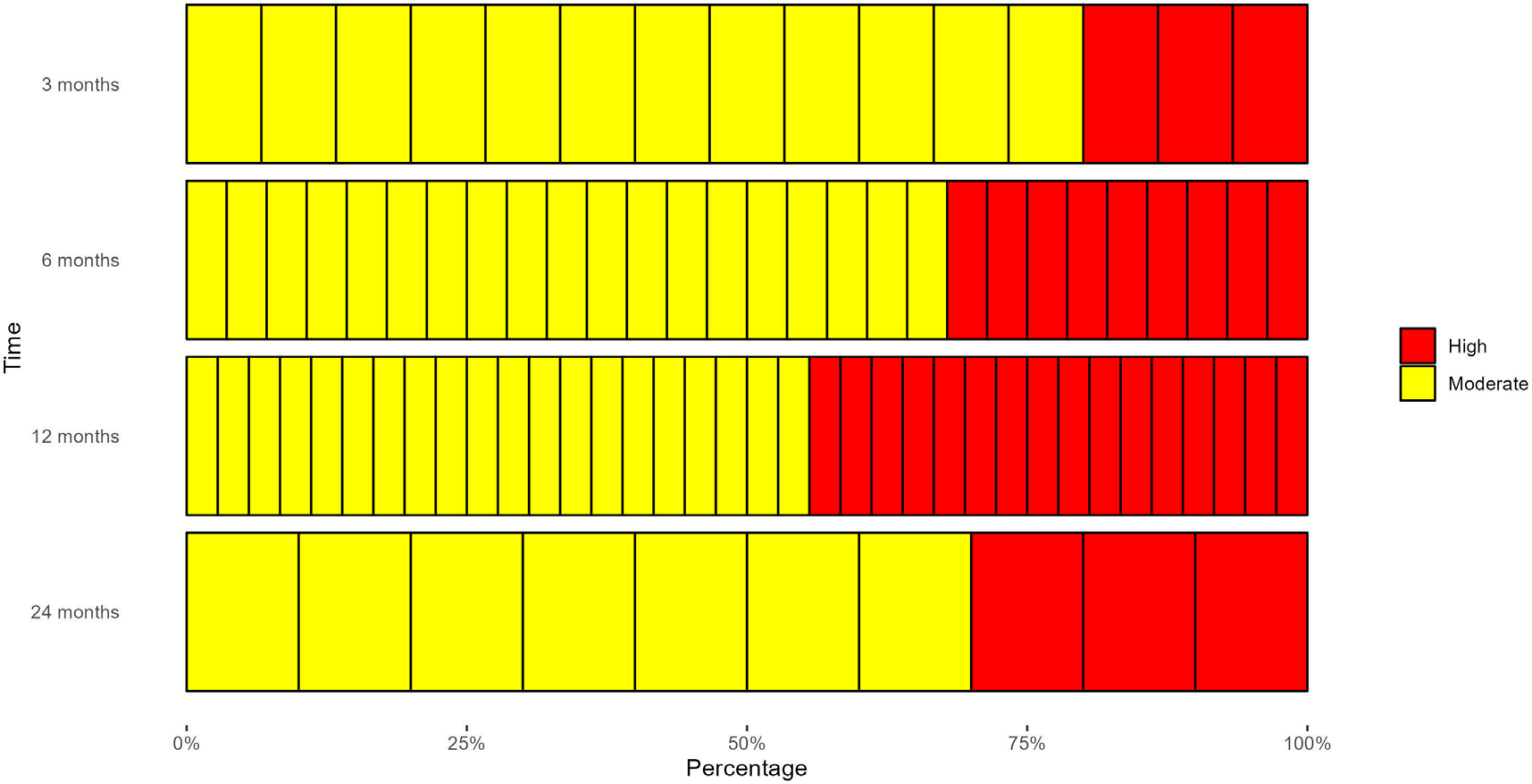
Summary of risk of bias assessments in TKR studies. Each block represents one study. Red represents an overall high risk of bias in a study; yellow represents an overall moderate risk of bias. TKR, total knee replacement.

After harmonising unfavourable pain outcomes at different time points, there were 15, 28, 36 and 10 studies with data available for 3, 6, 12 and 24 months postoperative. Risk of bias assessments are summarised in figure 2 (for traffic light plots, see online supplemental material S4). Most studies were judged as overall moderate risk of bias with few overall high risk of bias due to losses to follow-up of >25%, or use of scores which are not entirely patient completed or have concerns relating to a low pain cut-off.

**Figure 3:**
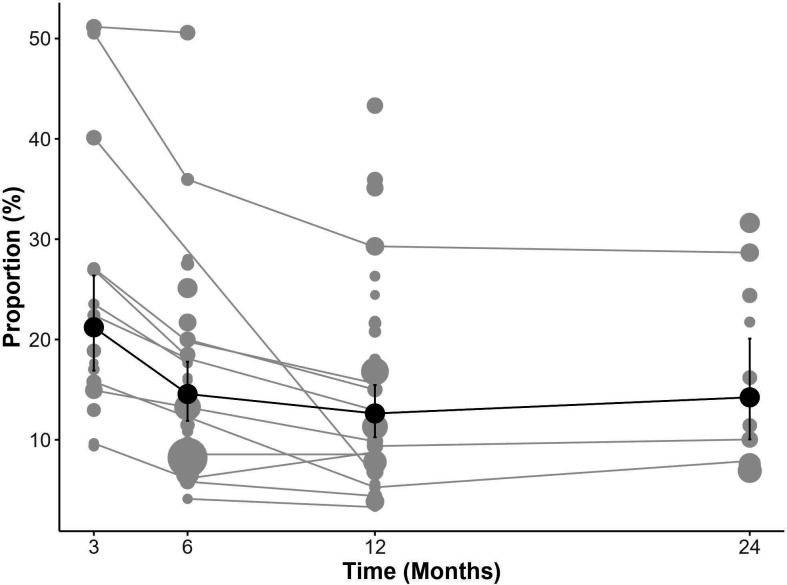
Multivariate meta-analysis of proportions over time in TKR studies plot. Grey dots and lines represent reported proportions across studies and time, while dark dots and lines show the multivariate meta-analysis results. The size of grey dots is proportional to the log of inverse variance. TKR, total knee replacement.

As noted in the previous review, the proportions of people with unfavourable pain varied widely across studies. Studies reported ranges of people with unfavourable pain at 3 months of 9.4%–51.2%, at 6 months of 4.1%–50.6%, at 12 months of 3.3%–43.3% and at 24 months of 6.9%–31.6% (online supplemental material S5). We synthesised the unfavourable pain outcomes using multivariate meta-analysis (figure 3), demonstrating a general decrease in pain proportions over time: 21.9% (95% CrI 15.6% to 29.4%) at 3 months, 14.1% (95% CrI 10.9% to 17.9%) at 6 months, 12.6% (95% CrI 9.9% to 15.9%) at 12 months and 14.6% (95% CrI 9.5% to 22.4%) at 24 months. The results of the univariate models were similar due to the limited number of studies with multiple time points (online supplemental material S5), though with slightly wider CrIs (online supplemental material S6). The substantial prediction intervals in the univariate models suggested considerable heterogeneity.

We investigated potential heterogeneity using meta-regression and subgroup analyses in the univariate meta-analysis models. Meta-regression results showed no evidence of age, percentage of women or sample size contributing to the heterogeneity of the proportion of individuals with unfavourable pain outcomes (online supplemental material S7).

Subgroup findings should be interpreted with caution due to the limited number of studies in some subgroups. In subgroup analyses, rates of unfavourable pain tended to be lower in studies involving patients from North America compared with other geographical groups (online supplemental material S8.1). Similarly, studies conducted in single-surgeon series settings showed lower rates of unfavourable pain outcomes (online supplemental material S8.2).

Outcome instruments that were not validated frequently suggested low levels of unfavourable pain, while multidimensional measures were consistent with overall meta-analysis at 3, 6, 12 and 24 months (online supplemental material S8.3). Results were also consistent for simple pain measures at 3, 6 and 12 months, but data were limited at 24 months. Cut-offs which defined an unfavourable pain outcome were based on pain intensity, symptom improvement, the functional impact of pain and minimally important clinical differences or patient acceptable symptom states calculated within each dataset. Excepting at 24 months when data were sparse, cut-offs relying on a simple dichotomisation by levels of pain intensity were reasonably consistent with meta-analyses (online supplemental material S8.4). In 3 and 5 studies, respectively, cut-offs based on minimally important clinical differences in WOMAC or KOOS outcomes at 6 and 12 months provided similar estimates of unfavourable pain to the meta-analyses. At 24 months, in three studies, the estimate of 10.88 (4.18 to 25.04) was lower than that in the overall meta-analysis, 14.6% (9.5% to 22.4%). Two studies reported the proportion of people not achieving a patient acceptable symptom state at 12 months. Results were similar to those in the overall meta-analysis. In the studies with cut-offs based on symptom improvement, the proportions of people with unfavourable pain were lower than seen in the overall meta-analyses.

Although we observed small-study effects in the results (online supplemental material S9), potentially attributable to publication bias, it is likely that these resulted from the extremely large variations in sample sizes at the 6, 12 and 24 months follow-ups. We did not find evidence of non-reporting bias, as most studies reported long-term pain outcomes in accordance with their reported methods.

In sensitivity analyses, we individually excluded studies with specific criteria to evaluate their impact on the univariate meta-analysis results (online supplemental material S10). The effects of excluding these studies were generally minor, except for studies with a high risk of bias or a high proportion of lost to follow-up. To account for the varying degrees of loss to follow-up, we performed separate scenario analyses by assuming that the same proportion of participants lost to follow-up experienced unfavourable pain outcomes in each study (table 2). By assuming 10%–30% of participants lost to follow-up might experience unfavourable pain, this approach could yield more realistic estimates, given the limited literature available for further imputation.

**Table 2:** Worst-best case scenario analyses in TKR studies

Proportion* (%)	Median (95% CrI)	τ² (95% CrI)
3 months		
0%	21.89 (15.72–29.35)	0.5 (0.19–1.1)
10%	23.8 (17.38–30.4)	0.4 (0.14–0.88)
20%	25.61 (19.46–32.34)	0.36 (0.12–0.78)
30%	27.22 (21–33.69)	0.31 (0.11–0.69)
40%	28.82 (22.45–35.25)	0.3 (0.1–0.66)
50%	30.68 (24.49–37.25)	0.27 (0.09–0.6)
60%	32.07 (25.66–38.42)	0.27 (0.09–0.6)
70%	33.55 (26.73–40.21)	0.28 (0.09–0.63)
80%	35.04 (28.15–41.98)	0.28 (0.1–0.63)
90%	36.71 (29.5–43.83)	0.3 (0.11–0.68)
100%	38.16 (30.6–45.68)	0.31 (0.11–0.69)
6 months		
0%	14.06 (10.79–17.79)	0.51 (0.26–0.88)
10%	16.37 (13.08–19.88)	0.37 (0.18–0.65)
20%	18.54 (15.24–22.09)	0.32 (0.16–0.56)
30%	20.5 (17.05–24.25)	0.3 (0.15–0.53)
40%	22.33 (18.66–26.38)	0.3 (0.15–0.52)
50%	24.22 (19.94–28.43)	0.32 (0.16–0.56)
60%	26.03 (21.65–30.67)	0.35 (0.18–0.6)
70%	27.91 (22.96–33.03)	0.39 (0.21–0.67)
80%	29.61 (24.15–35.12)	0.44 (0.23–0.75)
90%	31.39 (25.38–37.35)	0.51 (0.27–0.87)
100%	33.36 (26.84–40.12)	0.58 (0.31–1)
12 month		
0%	12.61 (9.88–15.84)	0.61 (0.34–0.97)
10%	15.22 (12.29–18.23)	0.44 (0.25–0.72)
20%	17.44 (14.5–20.66)	0.37 (0.2–0.6)
30%	19.6 (16.46–22.97)	0.36 (0.19–0.58)
40%	21.6 (18.09–25.17)	0.36 (0.2–0.58)
50%	23.6 (19.86–27.46)	0.37 (0.2–0.6)
60%	25.64 (21.74–29.89)	0.4 (0.23–0.64)
70%	27.57 (23.28–32.21)	0.44 (0.25–0.7)
80%	29.57 (24.55–34.51)	0.49 (0.28–0.78)
90%	31.53 (26.01–36.95)	0.55 (0.3–0.87)
100%	33.62 (27.69–39.61)	0.62 (0.37–0.99)
24 months		
0%	14.63 (8.83–21.5)	0.52 (0.15–1.32)
10%	16.67 (10.85–23.36)	0.41 (0.13–1.07)
20%	18.45 (12.81–25.31)	0.35 (0.11–0.91)
30%	20.23 (14.19–27.13)	0.34 (0.11–0.88)
40%	21.89 (15.29–29.1)	0.34 (0.11–0.88)
50%	23.64 (16.62–31.45)	0.35 (0.11–0.91)
60%	25.28 (17.78–33.83)	0.38 (0.12–0.97)
70%	26.89 (18.57–35.67)	0.4 (0.12–1.02)
80%	28.58 (19.92–38.38)	0.43 (0.14–1.11)
90%	30.04 (20.59–40.22)	0.48 (0.15–1.22)
100%	31.76 (21.49–42.8)	0.52 (0.15–1.32)

* Proportion: The proportion of lost to follow-up patients imputed to experience unfavourable pain outcomes.

CrI, credible interval.

### Total hip replacement

**Figure 4:**
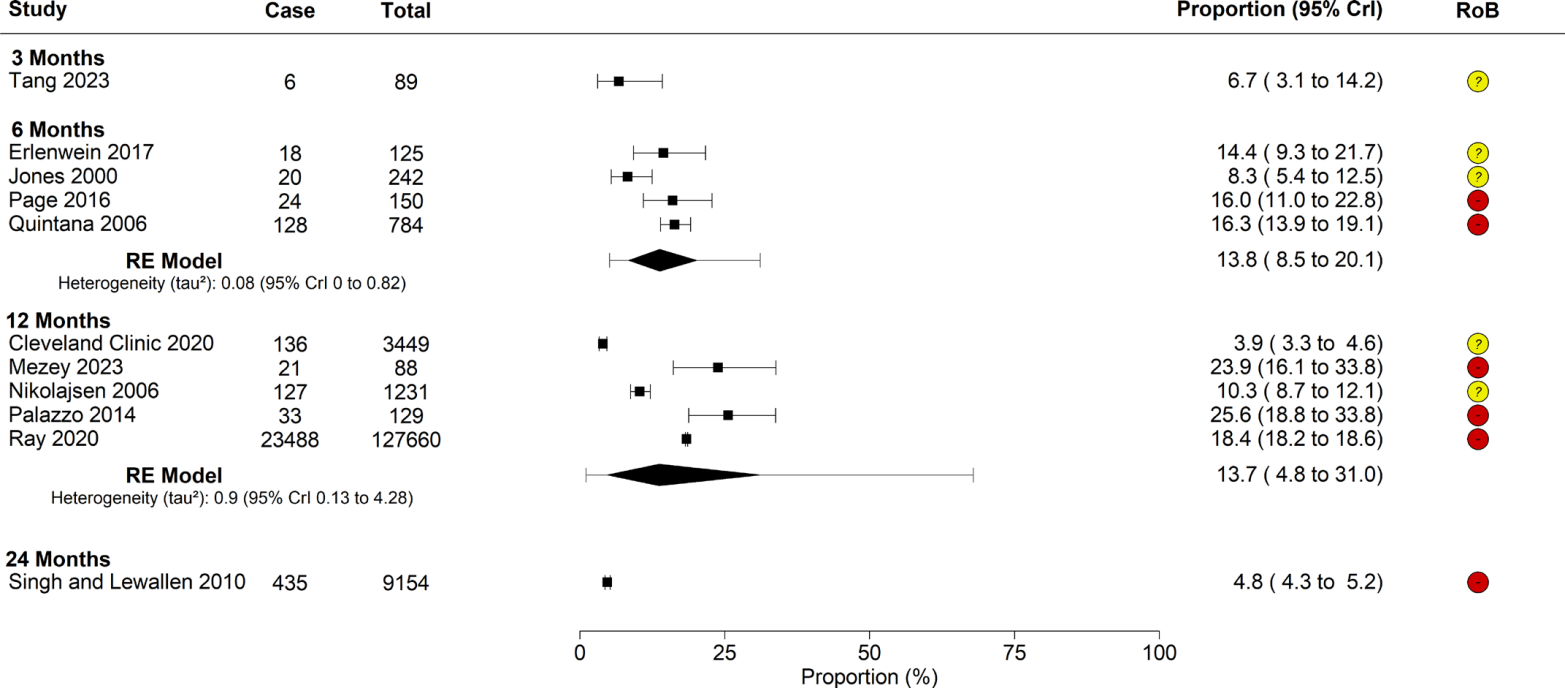
Forest plot of proportions over time in THR studies. Squares and bars represent the mean proportion of individual studies. Diamonds represent the point estimate and Crl of the meta-analysis results. The bars show the corresponding prediction intervals. Red circles and minus signs represent overall high risk of bias. Yellow circles and question marks represent overall moderate risk of bias. CrI, credible intervals; RE, random effects; RoB, risk of bias; THR, total hip replacement.

11 studies reported unfavourable pain outcomes in individuals who underwent THR. The characteristics of these studies are summarised in online supplemental material S11. Only one study reported unfavourable pain outcomes at the 3-month and 24-month time points, so a trend cannot be established. Studies reported ranges of people with unfavourable pain at 6 months of 8.3% to 16.3%, and at 12 months of 3.9% to 25.6% (figure 4).

Meta-analysis of unfavourable pain outcomes provided similar results at 6 and 12 months, with 13.8% (8.5% to 20.1%) and 13.7% (4.8% to 31.0%), respectively. However, concerns regarding the risk of bias assessment (online supplemental material S12) lead to low confidence in these results.

## Discussion

Through our systematic review and meta-analysis, we have synthesised the existing evidence on the proportion of patients who experience long-term pain after knee and hip replacement. By updating our previous review, we have been able to provide estimates of incidence rates at 3, 6, 12 and 24 months postoperative. As noted previously,^21^ studies report widely varying estimates of unfavourable pain outcome, and these may depend on the methods and analyses used. For example, at 12 months after TKR when patients should have recovered from surgery and be largely unaffected by issues relating to implant failure, the range of unfavourable pain across studies was 3.3%–43.3%. After THR at 12 months, the range was 3.9%–25.6%. With the large number of studies now available, meta-analyses have permitted us to provide point estimates with 95% CrI to describe uncertainty, and to explore patient and study-level factors that may explain the variation in unfavourable pain observed.

Our meta-analyses suggest that the proportion of people with an unfavourable level of pain after TKR decreases between three and 6 months after surgery and then remains stable until at least 2 years. While recognising the associated wide CrI, approximately 22% of patients will report an unfavourable pain outcome at 3 months after TKR, with 12%–15% of people experiencing an unfavourable longer-term pain outcome up to 2 years after surgery. For THR, a lack of studies reporting rates of unfavourable pain outcomes in unselected patients limited our analysis. However, our findings suggest that at least 14% of people may report unfavourable pain at 6–12 months after THR.

The strengths and limitations of this review should be considered when interpreting the results. First, the overall quality of evidence is low due to potential heterogeneity and risk of bias in TKR studies, and we were unable to estimate trends for THR studies due to a low number of included studies. Data from good quality registry studies were limited as estimates of proportions of people with chronic pain are seldom reported. The wide range of rates of unfavourable pain across studies may reflect the different definitions used by the study authors. However, we were unable to investigate conclusively the relationships between the definition used and prevalence estimates within this review as we did not have access to individual patient data. Studies in specific cohorts have reported proportions of people with different definitions of unfavourable pain outcomes.^18^ For example, in the study by Musbahi *et al*, thresholds based on combinations of different minimal clinically important differences and patient acceptable symptom states for WOMAC pain ranged from 5% to 52%.^18^ The authors note that a WOMAC pain score improvement of <20/100, as reported by 23% of people, had sensitivity and specificity for predicting a patient’s dissatisfaction with pain relief and overall outcome of TKR. We believe that studies reporting on different outcome assessments and those exploring the patient experience of pain after TKR and THR complement our research. The varying rates of unfavourable pain outcomes may also suggest that there is selection that was not apparent in the study methodology. For example, a single surgeon series with lower rates of unfavourable pain may relate to patient selection which is not evident from the cohort inclusion criteria. Second, loss to follow-up may have impacted on our estimates of the proportion of patients with chronic pain after TKR and THR. The influence that unfavourable pain and other outcomes have on patient willingness to participate in research follow-up is unclear. Some studies suggest that people with poor outcomes are less likely to participate in follow-up assessments due to dissatisfaction with their care or difficulties completing follow-up.^34^,^37^ However, others report no difference or poorer pain outcomes in those responding to initial invitations or attending follow-up visits compared with those not participating in follow-up visits.^38^,^40^ Our sensitivity analyses in studies of TKR excluding studies with high loss to follow-up rates showed higher rates of unfavourable pain and provided some support for the latter suggestion. Given the uncertainty regarding the impact of loss to follow-up, we conducted separate scenario analyses to provide readers with a range of realistic estimates for their consideration. Third, the scope of our review was broad. We included all different patient-reported measures of pain together, which present a mixture of single and multidimensional measures, and authors’ own definitions of unfavourable pain outcome. While this allowed us to take an encompassing approach to the synthesis of existing studies, it was likely an important source of heterogeneity in the results. It should also be noted that unfavourable pain does not necessarily equate with failure or dissatisfaction.^11^ Additionally, there were very few studies that provided multiple cut-off points for further analyses to elucidate the relationship between pain and satisfaction since the majority of studies only used a single postoperative VAS or NRS point. Additionally, it is also important to acknowledge that the included studies span over three decades, during which clinical practice and postoperative care may have evolved significantly. However, due to limited reporting and heterogeneity in study settings, designs and data collection periods, we were unable to formally explore the impact of temporal changes on the outcomes. Despite these limitations, this review is the most comprehensive attempt to date to collate the existing evidence and provides useful estimates to direct future research and improvements to clinical care.

Chronic pain after total knee or hip replacement has a highly negative impact on people^23 41^ to the extent that they may fear pursuing further healthcare and prescribed pain relief.^42^ For people who would potentially benefit from further care, how they are identified, assessed and treated varies considerably between centres in the UK.^43^ Cost implications for health services are considerable with numerous consultations, investigations and surgical referrals required.^44^ Chronic pain after joint replacement is an important research priority, as highlighted by the James Lind Alliance Priority Setting Partnership.^45^,^47^ Acknowledging that an estimated 13%–22% of people with TKR and a proportion of people with THR may experience chronic pain after surgery, implementation of evidence-based interventions aimed at the prevention and/or management of chronic pain after joint replacement is required.

Potential preoperative risk factors for chronic pain after total knee or hip replacement have been studied extensively with the aim of developing interventions and targeting care to those at risk. In a recent systematic review with 54 studies identified, there was no suggestion in meta-analyses that age, sex and body mass index were associated with the development of chronic pain after TKR.^48^ For a range of further potential risk factors including preoperative pain, evidence was limited with associations based on small numbers of studies or ‘vote counting’ analysis due to lack of data and methodological heterogeneity. For people receiving THR, consistent associations have been identified between female sex, high preoperative pain, poorer preoperative function, and anxiety or depression.^49 50^ Systematic reviews have identified that preoperative pain catastrophising, psychological distress and symptoms of anxiety and/or depression are risk factors for long-term pain after hip and knee replacement.^51^,^55^ Postoperative risk factors for chronic pain have been studied in TKR and largely relate to length of hospital stay, mechanical complications of the prosthesis, surgical site infection, hospital readmission, reoperation or revision,^56^ and patients with chronic pain are likely to undergo revision at a later time period.^57^ More generally, acute postoperative pain, caused by surgical methods and influenced by anaesthetic protocols, analgesia and care during the hospital admission, is also acknowledged as a risk factor for chronic postsurgical pain.^58 59^

There is a limited but growing body of evidence evaluating interventions that target risk factors for chronic pain after joint replacement.^60^,^63^ Preoperatively, general prehabilitation with exercise and education has not shown clear benefit for reduced long-term pain.^6060 64^,^67^ Another focus of efforts has been in removing delays to surgery to avoid possible decline in function and increase in pain while waiting for surgery. However, evidence of associations between longer waiting times for knee or hip replacement and chronic pain is equivocal.^68^,^70^ In randomised trials evaluating interventions targeting psychological risk factors, cognitive–behavioural therapy and pain coping skills programmes have not shown benefit for improved long-term pain.^6171^,^79^ However, a mindfulness-based stress-management intervention provided to patients before total hip or knee replacement surgery was associated with reduced long-term pain.^80^ During the perioperative period, the multimodal analgesia regimen provided may influence long-term pain outcomes, and there is some support for the incorporation of specific treatments, some of which are features of current pain management practice.^62 81^ After hospital discharge, care focuses mainly on physiotherapy-based rehabilitation, but there is no evidence to support one modality over another in relation to prevention of chronic pain.^63^ Exercise-based rehabilitation provided to people considered at risk of a poor outcome after TKR has shown little benefit for primary functional outcomes or long-term pain compared with usual care or less intensive interventions.^82 83^

Systematic reviews have identified a limited evidence base to guide the treatment and management of chronic pain after joint replacement and surgery more generally.^84 85^ To address this, a programme of research has been conducted focussing on the development and evaluation of an early postoperative intervention to prevent pain chronicity.^33^ Recognising the diverse causes of chronic pain, the STAR care pathway is a personalised and multifaceted intervention to reduce chronic pain after TKR.^86^ The care pathway involves the assessment of people with high levels of pain at 2–3 months after surgery to identify the underlying causes of pain with subsequent provision of referrals for appropriate treatment or management. Evaluation in a randomised controlled trial found the STAR care pathway was cost-effective and associated with a clinically important reduction in pain after 1 year compared with usual care.^86^ Furthermore, there is a suggestion of sustained benefit at up to 4 years.^87^

## Conclusions

The problem of chronic pain after knee and hip replacement is recognised by people who have pain, clinicians and the research community. Our review, bringing together all the published literature to date, suggests that a substantial proportion of patients continue to experience an unfavourable longer-term pain outcome up to 2 years after surgery. These findings highlight the need to improve pain management across the care pathway. There is an urgent need for the implementation of evidence-based interventions to optimise the management of chronic pain after joint replacement and evaluation of new preventive strategies that target established risk factors after joint replacement.

## Supplementary material

10.1136/bmjopen-2024-088975 online supplemental file 1

## Data Availability

Data are available on reasonable request.
