# Supplementary material for: What proportion of people have long-term pain after total hip or knee replacement? An update of a systematic review and meta-analysis
Source: BMJ Open. 2025 May 21;15(5):e088975. doi: 10.1136/bmjopen-2024-088975 (PMC12096998; doi:10.1136/bmjopen-2024-088975)

Supplementary materials

[S1. MOOSE checklist 3](#_Toc182396287)

[S2. Search Strategy as applied in MEDLINE and Embase 5](#_Toc182396288)

[S2.1 Total knee replacement 5](#_Toc182396289)

[S2.2 Total hip replacement 5](#_Toc182396290)

[S3. Characteristics of TKR studies 7](#_Toc182396291)

[References 14](#_Toc182396292)

[S3.1 Mean age and range 19](#_Toc182396293)

[S3.2 Proportion of females 19](#_Toc182396294)

[S3.3 Data collection timeframe 20](#_Toc182396295)

[S3.4 Proportions of lost to follow-ups and revisions 21](#_Toc182396296)

[S4. Traffic light plot of the risk of bias assessments in TKR studies 22](#_Toc182396297)

[S4.1 TKR studies (3 months) 22](#_Toc182396298)

[S4.2 TKR studies (6 months) 23](#_Toc182396299)

[S4.3 TKR studies (12 months) 24](#_Toc182396300)

[S4.4 TKR studies (24 months) 25](#_Toc182396301)

[S5. Forest plots of univariate meta-analyses in TKR studies 26](#_Toc182396302)

[S5.1 TKR studies (3 months) 26](#_Toc182396303)

[S5.2 TKR studies (6 months) 26](#_Toc182396304)

[S5.3 TKR studies (12 months) 27](#_Toc182396305)

[S5.4 TKR studies (24 months) 27](#_Toc182396306)

[S6. Table of multivariate and univariate meta-analysis results in TKR studies 28](#_Toc182396307)

[S7. Meta-regression analyses in TKR studies 29](#_Toc182396308)

[S7.1 Mean age 29](#_Toc182396309)

[S7.2 Proportion of females 29](#_Toc182396310)

[S7.3 Sample sizes 29](#_Toc182396311)

[S8. Subgroup analyses in TKR studies 30](#_Toc182396312)

[S8.1 Geographical regions 30](#_Toc182396313)

[S8.2 Setting 30](#_Toc182396314)

[S8.3 Pain outcome instruments 31](#_Toc182396315)

[S8.4 Cut-off definitions 31](#_Toc182396316)

[S9. Doi plots and the LFK indexes in TKR studies 33](#_Toc182396317)

[S9.1 TKR studies (3 months) 33](#_Toc182396318)

[S9.2 TKR studies (6 months) 33](#_Toc182396319)

[S9.3 TKR studies (12 months) 34](#_Toc182396320)

[S9.4 TKR studies (24 months) 34](#_Toc182396321)

[S10. Sensitivity analyses 35](#_Toc182396322)

[S11. Characteristics of THR studies 36](#_Toc182396323)

[References 37](#_Toc182396324)

[S11.1 Proportions of lost to follow-ups and revisions 39](#_Toc182396325)

[S12. Traffic light plot of the risk of bias assessments in THR studies 40](#_Toc182396326)

# S1. MOOSE checklist

| Item No | Recommendation | Reported on Page Number | Reported on Section/Paragraph |
| --- | --- | --- | --- |
| Reporting of background | | | |
| 1 | Problem definition | Page 4 | Introduction |
| 2 | Hypothesis statement | Page 4 | Introduction |
| 3 | Description of Study Outcome(s) | Page 5 | Outcome, Methods |
| 4 | Type of exposure or intervention used | Page 5 | Eligibility Criteria, Methods |
| 5 | Type of study design used | Page 5 | Eligibility Criteria, Methods |
| 6 | Study population | Page 5 | Eligibility Criteria, Methods |
| Reporting on search strategy | | | |
| 7 | Qualifications of searchers (e.g., librarians and investigators) | Using existing search strategies |  |
| 8 | Search strategy, including time period included in the synthesis and keywords |  | Supplementary S2 |
| 9 | Effort to include all available studies, including contact with authors | Page 5-6 | Searches, Methods |
| 10 | Databases and registries searched | Page 5 | Searches, Methods |
| 11 | Search software used, name and version, including special features used (e.g., explosion) | Page 6 | Study selection and data collection, Methods |
| 12 | Use of hand searching (e.g., reference lists of obtained articles) | Page 5 | Searches, Methods |
| 13 | List of citations located and those excluded, including justification |  | Figure 1 (PRISMA flowchart) |
| 14 | Method for addressing articles published in languages other than English | Page 5 | Searches, Methods |
| 15 | Method of handling abstracts and unpublished studies | Page 5 | Searches, Methods |
| 16 | Description of any contact with authors | Page 5-6 | Searches, Methods |
| Reporting of methods | | | |
| 17 | Description of relevance or appropriateness of studies assembled for assessing the hypothesis to be tested |  | Table 1 |
| 18 | Rationale for the selection and coding of data (e.g., sound clinical principles or convenience) | Page 6 | Study selection and data collection, Methods |
| 19 | Documentation of how data were classified and coded (e.g., multiple raters, blinding, and interrater reliability) | Page 6 | Study selection and data collection, Methods |
| 20 | Assessment of confounding (e.g., comparability of cases and controls in studies where appropriate) |  | Tables 1, S3 and S11 |
| 21 | Assessment of study quality, including blinding of quality assessors; stratification or regression on possible predictors of study results | Page 6 | Risk of bias assessment/ Methods |
| 22 | Assessment of heterogeneity | Page 7 | Exploration of heterogeneity, Methods |
| 23 | Description of statistical methods (e.g., complete description of fixed or random effects models, justification of whether the chosen models account for predictors of study results, dose-response models, or cumulative meta-analysis) in sufficient detail to be replicated | Page 6-7 | Data synthesis approach, Methods |
| 24 | Provision of appropriate tables and graphics |  | Results |
| Reporting of results | | | |
| 25 | Graphic summarizing individual study estimates and overall estimate |  | Figures 3, 4, and S5 |
| 26 | Table giving descriptive information for each study included |  | Tables S3 and S11 |
| 27 | Results of sensitivity testing (e.g., subgroup analysis) |  | Tables S7, S8, and S10 |
| 28 | Indication of statistical uncertainty of findings |  | Table 2 and S6 |
| Reporting of discussion | | | |
| 29 | Quantitative assessment of bias (e.g., publication bias) |  | Figures S9 |
| 30 | Justification for exclusion (e.g., exclusion of non–English-language citations) |  | Not applicable |
| 31 | Assessment of quality of included studies |  | Figures 2 and 4 |
| Reporting of conclusions | | | |
| 32 | Consideration of alternative explanations for observed results |  | Conclusion |
| 33 | Generalization of the conclusions (i.e., appropriate for the data presented and within the domain of the literature review) |  | Conclusion |
| 34 | Guidelines for future research |  | Conclusion |
| 35 | Disclosure of funding source |  | Sources of funding |

*From:*  Brooke BS, Schwartz TA, Pawlik TM. MOOSE Reporting Guidelines for Meta-analyses of Observational Studies. JAMA Surg. 2021;156(8):787–788. doi:10.1001/jamasurg.2021.0522

# S2. Search Strategy as applied in MEDLINE and Embase

## S2.1 Total knee replacement

*Medline*

1. survey.mp. or exp Data Collection/

2. prospective study.mp. or exp Prospective Studies/

3. observational study.mp.

4. exp EPIDEMIOLOGY/ or epidemiology.mp.

5. longitudinal study.mp. or exp Longitudinal Studies/

6. follow up study.mp. or exp Follow-Up Studies/

7. exp Arthroplasty, Replacement, Knee/ or exp Knee Prosthesis/ or knee replacement.mp.

8. knee prosthesis.mp. or exp Knee Prosthesis/

9. total knee.tw.

10. (knee adj10 (replace$ or arthroplast$ or prosthe$ or implant$)).ti, ab.

11. 7 or 8 or 9 or 10

12. pain.tw.

13. 1 or 2 or 3 or 4 or 5 or 6

14. 10 and 12 and 13

*Embase*

1. Clinical study/

2. Longitudinal study/

3. Prospective study/

4. Cohort analysis/

5. (Cohort adj (study or studies)).mp.

6. (follow up adj (study or studies)).tw.

7. (observational adj (study or studies)).tw.

8. (epidemiologic$ adj (study or studies)).tw.

9. exp Arthroplasty, Replacement, Knee/ or exp Knee Prosthesis/ or knee replacement.mp.

10. knee prosthesis.mp. or exp Knee Prosthesis/

11. total knee.tw.

12. (knee adj10 (replace$ or arthroplast$ or prosthe$ or implant$)).ti,ab.

13. pain.tw.

14. 1 or 2 or 3 or 4 or 5 or 6 or 7 or 8

15. 9 or 10 or 11 or 12

16. 13 and 14 and 15

## S2.2 Total hip replacement

*Medline*

1. survey.mp. or exp Data Collection/

2. prospective study.mp. or exp Prospective Studies/

3. observational study.mp.

4. exp EPIDEMIOLOGY/ or epidemiology.mp.

5. longitudinal study.mp. or exp Longitudinal Studies/

6. follow up study.mp. or exp Follow-Up Studies/

7. exp Arthroplasty, Replacement, Hip/ or exp Hip Prosthesis/ or hip replacement.mp.

8. hip prosthesis.mp. or exp hip Prosthesis/

9. total hip.tw.

10. (hip adj10 (replace$ or arthroplast$ or prosthe$ or implant$)).ti, ab.

11. 7 or 8 or 9 or 10

12. pain.tw.

13. 1 or 2 or 3 or 4 or 5 or 6

14. 10 and 12 and 13

*Embase*

1. Clinical study/

2. Longitudinal study/

3. Prospective study/

4. Cohort analysis/

5. (Cohort adj (study or studies)).mp.

6. (follow up adj (study or studies)).tw.

7. (observational adj (study or studies)).tw.

8. (epidemiologic$ adj (study or studies)).tw.

9. exp Arthroplasty, Replacement, hip/ or exp hip Prosthesis/ or hip replacement.mp.

10. hip prosthesis.mp. or exp hip Prosthesis/

11. total hip.tw.

12. (hip adj10 (replace$ or arthroplast$ or prosthe$ or implant$)).ti,ab.

13. pain.tw.

14. 1 or 2 or 3 or 4 or 5 or 6 or 7 or 8

15. 9 or 10 or 11 or 12

16. 13 and 14 and 15

# S3. Characteristics of TKR studies

| **Study**  **Country**  **Recruitment dates**  **Setting** | **Operation**  **Number of patients**  **Age (SD), range**  **% women** | **Pain measure** | **Definition of unfavourable pain outcome**  **High risk of bias concern** |
| --- | --- | --- | --- |
| Alzahrani 2011[1] TWH cohort  Canada  1998-2007  2 hospitals | Primary TKR, all 18+  N=482  67.5 (9.6)  62% | WOMAC pain  12 months | No clinically important improvement based on MCID (WOMAC index of 7.5) |
| Aso 2020[2]  Japan  2012-2017  1 hospital | Primary TKR, all  N=234  75  75.8% | VAS/NRS pain  6, 12 months | Moderate to severe pain (VAS >30 mm), at rest or walking |
| Attal 2014[3]  France  2008-2011  I hospital | Primary TKR, all 18+  N=89  68.7 (8.9)  65.0% | BPI (NRS)  3, 6, 12 months | NRS pain average 3 or greater on 10-point scale |
| Baker 2007[4]  UK  2003  National registry | Primary TKR, all  N=9417  70.68  56.8% | OKS pain  12 months | Reported persistent knee pain |
| Bell 2023[5]  USA  2015-2018  7 hospitals | Primary TKR, all 50-89  N=5564  Range 50-89  60.7% | KOOS pain  12 months | MCID not satisfied (15 points) |
| Birch 2019[6]  Denmark  2011-2013  1 hospital | Primary TKR or UKR, all  N=589  67.3 (9.7)  52.0% | OKS pain  4, 12 months | OKS pain moderate/severe  High loss to follow up rate at 4 and 12 months |
| Brander 2003[7]  USA  1998-2000  1 surgeon | Primary TKR, all 18+  N=116  66 (10.5), range 36-85  55.2% | VAS/NRS pain  3, 6, 12 months | VAS >40 |
| Buus 2022[8]  Denmark  2015-2016  1 hospital | Primary TKR, all 18+  N=217  66.8 (9.3)  52.2% | OKS pain  12 months | Threshold 42.39[69] |
| Buvanendran 2019[9]  USA  2011-2017  1 hospital | Primary TKR, all  N=296  65  65.3% | VAS/NRS pain  6 months | NRS pain with movement ≥4 |
| Chodor and Kruczynski 2022[10]  Poland  2016  1 hospital | Primary TKR, all 48+  N=69  67.6 (7.42), range 48-84  76.7% | Author own question  6 months | Pain severely limiting daily life |
| Clement 2014[11]  UK  2010  1 hospital | Primary TKR, all  N=578  70 (9.6), range 39-91  58.4% | Author’s own question “How well did the surgery relieve pain in your affected joint?”  12 months | Fair or poor  High loss to follow up rate |
| Cole 2022[12]  UK  2010-2015  2 hospitals | Primary TKR, all  N=1025  70  55.8% | OKS pain  12 months | <14 points OKS |
| Dave 2017[13]  USA  2012-2014  3 hospitals | Primary TKR probably, all 40+  N=267  66 (9)  61.0% | WOMAC pain  12 months | WOMAC pain score < MCID (WOMAC pain of 15) |
| Dowsey 2012[14]  Australia  2006-2007  1 hospital | Primary TKR, all  N=478  70.8 (8.3), range 45-90  69.2% | IKSS pain  12, 24 months | IKSS pain score <30 moderate to severe pain  IKSS may not be entirely patient reported at 12 and 24 months |
| Dursteler 2021[15]  Spain  2014-2017  Spain  1 hospital | Primary TKR, all 18+  N=170  73.1 (7.1)  73.3% | VAS/NRS pain  3, 6 months | NRS 0.3/1 or greater at rest |
| Edwards 2022[16]  USA  2012-2018  2 hospitals | Primary TKR, all 45+  N=248  65.1 (8.2)  59.5% | BPI  6 months | 4/10 or greater  High loss to follow up rate |
| Escobar and Riddle 2014[17]  Spain  2003-2006  15 hospitals | Primary TKR, all  N=1616  71.6 (6.8)  70.0% | WOMAC pain  12 months | Number not attaining PASS (i.e. “No” in the question, “*If you had to be the rest of your life with the symptoms you have now, how would you feel?*”) as the twenty-fifth percentile of the final score at 1 year instead of the seventy-fifth percentile (reverse option for WOMAC scores).  High loss to follow up rate |
| Getachew 2021[18]  Norway  2012-2014  1 hospital | Primary TKR, all 18+  N=206  68 (9)  66.0% | BPI  12 months | BPI worst pain score ≥4 |
| Ghomrawi 2017[19]  USA  2010-2012  1 hospital | Primary TKR, all  N=247  68 (10)  65.0% | WOMAC pain  24 months | Number not achieving MCID (baseline-adjusted MCIDs, as described by Escobar et al.[70]) |
| Grosu 2016[20]  Belgium  2009-2010  1 surgeon | Primary TKR probably, all  N=114  66 (10)  65.8% | VAS/NRS pain  3, 6, 12 months | Moderate to severe pain  High loss to follow up rate at 3, 6 and 12 months |
| Hardy 2022[21]  France  2014-2015  1 hospital | Primary TKR, all >18  N=111  73.3 (9.3) range 29-92  65.0% | VAS/NRS pain  12 months | VAS >30/100 |
| Heath 2021[22]  Australia  2018-2020  44 hospitals | Primary and revision TKR, all  N=8299  67.5 (8.8)  56.4% | EQ-5D 5L pain/ discomfort  6 months | Moderate/ severe or extreme pain EQ 5D 5L pain/discomfort  High loss to follow up rate |
| Jones 2000[23]  Canada  1995-1997  1 health region | Primary TKR, all 40+  N=292  69.2 (9.2)  59.0% | WOMAC pain  6 months | Moderate/ severe pain defined as a gain of <10 points on the WOMAC pain dimension |
| Khalid 2021[24]  UK  2008-2016  National registry | Primary TKR or UKR, all  N=531,790  69.7 (9.4)  56.6% | OKS pain  6 months | OKS-pain score of 14 or less at six months after knee replacement can be considered to be in chronic pain |
| Kim 2015[25]  South Korea  2013-2014  1 hospital | Primary TKR, all women  N=94  70.18 (5.74), range 20-80  100% | VAS/NRS pain  3 months | >5 points on an 11 point VAS/NRS (verbal numeric rating scale) |
| Kiran 2015[26]  UK  2003-2007  1 hospital | Primary TKR, all  N=608  72  61.4% | OKS pain  12, 24 months | Has your knee replacement operation decreased your knee pain?  High loss to follow up rate at 12 and 24 months |
| Kornilov 2018[27]  Russia  2014  1 hospital | Primary TKR, all 18+  N=100  63 (8), range 47-81  95.0% | VAS/NRS pain  12 months | Not at least a two-point or approximately 30% (clinically significant) decrease in rating of pain interference with walking from baseline to 1 year (NRS scale 0-10) |
| Kurien 2018[28]  UK  Before 2017  1 hospital | Primary TKR probably, all  N=50  66.4 (8.3)  60.0% | VAS/NRS pain  6 months | 4 or greater |
| Larsen 2021[29]  Denmark  2015-2016  1 hospital | Primary TKR, all 18+  N=185  68.8 (8.9)  55.7% | VAS/NRS pain  12 months | Pain intensity at rest >3  High loss to follow up rate |
| Latijnhouwers 2022[30]  The Netherlands  2012-2017  2 hospitals | Primary TKR, all  N=282  66 (8.4)  63.0% | VAS/NRS pain  12 months | Moderate to severe pain (NRS ≥4)  High loss to follow up rate |
| Lavand’homme 2014[31]  Belgium  2012  1 surgeon | Primary TKR or UKR, all  N=128  68 (10)  66.4% | VAS/NRS pain  3 months | NRS ≥4/10 |
| Lee 2022[32]  South Korea  2017-2019  2 surgeons | Primary TKR probably, all  N=172  70.7 (4.3)  89.2% | Pain disturbing sleep  3, 12 months | Night pain was defined as pain around the knee experienced at night that could disturb the patient’s sleep |
| Leppanen 2021[33]  Finland  2012-2014  1 hospital | Primary TKR, 65 years or younger  N=205  60  63.0% | VAS pain exercise  24 months | VAS >30 |
| Leung 2019[34]  Singapore  2015  1 hospital | Primary TKR, all  N=243  66 (8.3)  78.6% | Author own question  6, 12 months | No change or worsening pain/ slightly better |
| Lundblad 2008[35]  Sweden  Before 2006  1 hospital | Primary TKR, all  N=69  68  50.7% | VAS/NRS pain  24 months | Pain at rest, VAS >2/10 |
| Lyman 2018[36]  USA  2007-2012  1 hospital | Primary TKR, all  N=3815  74 (6)  63.0% | KOOS pain  24 months | Number not achieving MCID (8 by distribution-based method [71])  High loss to follow up rate |
| Mahdi 2020[37]  Sweden  2016-2018  3 hospitals | Primary TKR, all  N=615  69.7  52.2% | KOOS pain  12 months | 8 cut off  High loss to follow up rate |
| Mekkawy 2023[38]  USA  2021  4 surgeons | Primary TKR, all  N=112  65.5 (9.2)  69.0% | VAS pain  6 months | Probably NRS score of ≥1 in defined sites  Concern over VAS ≥1 being too inclusive and high loss to follow up rate |
| Mercurio 2020[39]  Italy  2015-2017  1 hospital | Primary TKR, all >18  N=45  69.6 (7.8)  65.0% | VAS/NRS pain  12 months | VAS >30 residual pain |
| Mezey 2023[40]  Hungary  2019-2020  2 hospitals | Primary TKR probably, all  N=101  69.2  Not reported | WOMAC pain  12 months | Not exceeding MCID (WOMAC pain of 13.3)  High loss to follow up rate |
| Musbahi 2023[41]  USA  2011-2014  4 hospitals | Primary TKR, all 40+  N=575  66.3 (8.3)  60% | WOMAC pain  12 months | WOMAC pain score (converted to a 0-to-100 scale) improvement of <20  High loss to follow up rate |
| Nishimoto 2023[42]  Japan  2021-2023  1 hospital | Primary TKR, all with no complications  N=68  75.1 (7.3)  80.9% | KOOS pain  3, 6 months | Not achieving MCID of 10 (3 months) and 13 (6 months). MCID was calculated using the anchor method.[72] |
| Noiseux 2014[43]  USA  Before 2012  2 hospitals | Primary TKR, all 30+  N=215  61.7 (9.8)  58.0% | VAS/NRS pain  6 months | Moderate or severe pain with range of motion, VAS ≥1  Concern over VAS ≥1 being too inclusive |
| Orr 2022[44]  USA  2016-2019  9 hospitals | Primary TKR, all  N=7476  67 (9.0)  60.8% | KOOS pain  12 months | Not achieved PASS (i.e. “No” in the question, “*Taking into account all the activity you have during your daily life, your level of pain and also your activity limitations and participation restrictions, do you onsider the current state of your*  *knee satisfactory?*”) for KOOS pain  High loss to follow up rate |
| Petersen 2015[45]  Denmark  Before 2014  1 hospital probably | Primary TKR, all  N=78  69  59.0% | VAS/NRS pain  12 months | VAS >3  High loss to follow up rate |
| Petersen 2018[46]  Denmark  Before 2017  1 hospital | Primary TKR probably, all  N= 200  69 (1.2)  57.0% | VAS/NRS pain  12 months | <30% reduction in pain |
| Phillips 2014[47]  UK  2009-2010  1 hospital | Primary TKR, all  N= 96  70.6  56.0% | VAS/NRS pain  3, 6, 12 months | VAS >3 |
| Priol 2023[48]  France  2011-2012  1 hospital | Primary TKR, all  N=129  74 (10), range 45-94  72.3% | VAS/NRS pain  6 months | VAS 4+  High loss to follow up rate |
| Pua 2019[49]  Singapore  2013-2017  1 hospital | Primary TKR, all 50+  N=5325  68 (7.5)  75.0% | OKS pain  6 months | Moderate or severe pain |
| Quintana 2006[50]  Spain  1999-2000  7 hospitals | Primary TKR, all  N=792  71.9  73.0% | WOMAC pain  6 months | No improvement in pain greater than MCID (22.60 of 100) using an anchor-based method. |
| Rice 2018[51]  New Zealand  2012-2015  3 hospitals | Primary TKR, all 18+  N=300  69 (10), range 48-90  48.0% | VAS/NRS pain  6, 12 months | VAS >3 |
| Sideris 2022[52]  USA  2016-2018  1 hospital | Primary TKR, all  N=179  67.1 (8.1)  56.2% | VAS/NRS pain  6 months | NRS 4+ |
| Singh 2014[53]  USA  1993-2005  1 hospital | Primary TKR, all  N=7229  68 (10)  56.0% | Author own question  24 months | Moderate-severe pain |
| Solberg 2023[54]  USA  2020  22 surgeons | Primary TKR probably, all  N=239  66.2 (8.5), range 37-87  60.7% | Author own question  3 months | To what extent have you obtained relief: somewhat, minimal or not at all  High loss to follow up rate |
| Stephens 2002[55]  USA  Before 2001  1 hospital | Primary TKR, all 50+  N=68  67.4 (8.1), range 50-88  54.0% | WOMAC pain  6 months | No change or increase in pain from pre-operative |
| Tang 2023[56]  China  2020-2021  1 hospital | Primary TKR probably, all 65+  N=196  72  75.1% | VAS/NRS pain  3 months | NRS scores ≥4 |
| Terradas-Monllor 2024[57]  Spain  2018-2020  1 home rehabilitation service | Primary TKR or UKR, all 18+  N=115  70.5 (10.7)  66.1% | VAS/NRS pain  3, 6 months | VAS 3+ |
| Thomazeau 2016[58]  France  2013  1 hospital | Primary TKR, all  N=109  69.2 (9)  71.6% | VAS/NRS pain  6 months | NRS score ≥1/10 for the last 8 days |
| Tian 2022[59]  China  2018-2019  1 hospital | Primary TKR or UKR, all <90  N=271  Not reported  80.8% | Author own question  24 months | Moderate or severe pain on movement |
| Utrillas-Compaired 2014[60]  Spain  2009  1 hospital | Primary TKR, all  N=215  73 (6.35)  69.3% | KSS pain  12 months | KSS pain poor (less than 60 points)  KSS may not be entirely patient reported |
| van der Wees 2017[61]  The Netherlands  1993-2014  1 hospital | Primary TKR, all  N=704  65 (12)  64.5% | VAS/NRS pain  6, 12 months | 30% or less improvement in VAS pain  High loss to follow up rate at 6 and 12 months |
| Vina 2020[62]  USA  2005-2015  4 hospitals | Primary TKR, all  N=315  67.3 (8.6)  60.9% | WOMAC pain  24 months | Less than MCID of 1.5 |
| Vuorenmaa 2008[63]  Finland  Before 2007  2 surgeons | Primary TKR, all <80  N=51  70 (5)  80% | VAS/NRS pain  3 months | VAS >30/100 |
| W-Dahl 2014[64]  Sweden  2008-2010  2 hospitals | Primary TKR, all  N=2736  69.3 (8.7)  58.5% | KOOS pain  12 months | Unchanged or worse pain |
| Waimann 2014[65]  USA  2004-2007  2 hospitals | Primary TKR, all  N=236  65.1 (8.9)  66.0% | WOMAC pain  6 months | Less than MCID of ≥20 in both the WOMAC pain and function scores (scaled to 100) |
| Wylde 2013[66]  UK  2010-2011  1 hospital | Primary TKR, all  N=57  68  58% | WOMAC pain  12 months | WOMAC pain score of >75 |
| Wylde 2019[67]  UK  2006-2009  1 hospital | Primary TKR, all eligible for Triathlon prosthesis  N=266  70 (9.9), range 41-90  64% | WOMAC pain  3, 12, 24 months | Worse or no change in WOMAC pain of 14 point (based on MCID)  High loss to follow up rate |
| Yan 2023[68]  China  2021-2023  1 hospital | Primary TKR, all 45+  N=470  63.4 (7.4)  69.9% | VAS/NRS pain  6 months | NRS score of ≥1 at rest and/or on movement  Concern over VAS ≥1 being too inclusive |

*Abbreviations: PASS (Patient Acceptable Symptom State), MCID (Minimal Clinically

Important Difference)

## References

1. Alzahrani K, Gandhi R, Debeer J, Petruccelli D, Mahomed N. Prevalence of clinically significant improvement following total knee replacement. J Rheumatol. 2011;38(4):753-9.

2. Aso K, Ikeuchi M, Takaya S, Sugimura N, Izumi M, Wada H, Okanoue Y, Dan J. Chronic postsurgical pain after total knee arthroplasty: A prospective cohort study in Japanese population. Mod Rheumatol. 2021;31(5):1038-44.

3. Attal N, Masselin-Dubois A, Martinez V, Jayr C, Albi A, Fermanian J, Bouhassira D, Baudic S. Does cognitive functioning predict chronic pain? Results from a prospective surgical cohort. Brain. 2014;137(Pt 3):904-17.

4. Baker PN, van der Meulen JH, Lewsey J, Gregg PJ, National Joint Registry for E, Wales. The role of pain and function in determining patient satisfaction after total knee replacement. Data from the National Joint Registry for England and Wales. J Bone Joint Surg Br. 2007;89(7):893-900.

5. Bell JA, Emara AK, Barsoum WK, Bloomfield M, Briskin I, Higuera C, Klika AK, Krebs VE, Mesko NW, Molloy RM, Mont MA, Murray TG, Muschler GF, Nickodem RJ, Patel PD, Schaffer JL, Stearns KL, Strnad GJ, Piuzzi NS. Should an Age Cutoff Be Considered for Elective Total Knee Arthroplasty Patients? An Analysis of Operative Success Based on Patient-Reported Outcomes. J Knee Surg. 2023;36(9):1001-11.

6. Birch S, Stilling M, Mechlenburg I, Hansen TB. The association between pain catastrophizing, physical function and pain in a cohort of patients undergoing knee arthroplasty. BMC Musculoskelet Disord. 2019;20(1):421.

7. Brander VA, Stulberg SD, Adams AD, Harden RN, Bruehl S, Stanos SP, Houle T. Predicting total knee replacement pain: a prospective, observational study. Clin Orthop Relat Res. 2003(416):27-36.

8. Buus AAO, Udsen FW, Laugesen B, El-Galaly A, Laursen M, Hejlesen OK. Patient-Reported Outcomes for Function and Pain in Total Knee Arthroplasty Patients. Nurs Res. 2022;71(5):E39-E47.

9. Buvanendran A, Della Valle CJ, Kroin JS, Shah M, Moric M, Tuman KJ, McCarthy RJ. Acute postoperative pain is an independent predictor of chronic postsurgical pain following total knee arthroplasty at 6 months: a prospective cohort study. Reg Anesth Pain Med. 2019;44(3):287-96.

10. Chodor P, Kruczynski J. Preoperative Risk Factors of Persistent Pain following Total Knee Arthroplasty. Biomed Res Int. 2022;2022:4958089.

11. Clement ND, MacDonald D, Simpson AH. The minimal clinically important difference in the Oxford knee score and Short Form 12 score after total knee arthroplasty. Knee Surg Sports Traumatol Arthrosc. 2014;22(8):1933-9.

12. Cole S, Kolovos S, Soni A, Delmestri A, Sanchez-Santos MT, Judge A, Arden NK, Beswick AD, Wylde V, Gooberman-Hill R, Pinedo-Villanueva R. Progression of chronic pain and associated health-related quality of life and healthcare resource use over 5 years after total knee replacement: evidence from a cohort study. BMJ Open. 2022;12(4):e058044.

13. Dave AJ, Selzer F, Losina E, Usiskin I, Collins JE, Lee YC, Band P, Dalury DF, Iorio R, Kindsfater K, Katz JN. The association of pre-operative body pain diagram scores with pain outcomes following total knee arthroplasty. Osteoarthritis Cartilage. 2017;25(5):667-75.

14. Dowsey MM, Nikpour M, Dieppe P, Choong PF. Associations between pre-operative radiographic changes and outcomes after total knee joint replacement for osteoarthritis. Osteoarthritis Cartilage. 2012;20(10):1095-102.

15. Dursteler C, Salazar Y, Rodriguez U, Pelfort X, Verdie LP. Conditioned pain modulation predicts persistent pain after knee replacement surgery. Pain Rep. 2021;6(1):e910.

16. Edwards RR, Campbell C, Schreiber KL, Meints S, Lazaridou A, Martel MO, Cornelius M, Xu X, Jamison RN, Katz JN, Carriere J, Khanuja HP, Sterling RS, Smith MT, Haythornthwaite JA. Multimodal prediction of pain and functional outcomes 6 months following total knee replacement: a prospective cohort study. BMC Musculoskelet Disord. 2022;23(1):302.

17. Escobar A, Riddle DL. Concordance between important change and acceptable symptom state following knee arthroplasty: the role of baseline scores. Osteoarthritis Cartilage. 2014;22(8):1107-10.

18. Getachew M, Lerdal A, Smastuen MC, Gay CL, Aamodt A, Tesfaye M, Lindberg MF. High levels of preoperative pain and fatigue are red flags for moderate-severe pain 12 months after total knee arthroplasty-A longitudinal cohort study. Musculoskeletal Care. 2021;19(2):186-92.

19. Ghomrawi HMK, Mancuso CA, Dunning A, Gonzalez Della Valle A, Alexiades M, Cornell C, Sculco T, Bostrom M, Mayman D, Marx RG, Westrich G, O'Dell M, Mushlin AI. Do Surgeon Expectations Predict Clinically Important Improvements in WOMAC Scores After THA and TKA? Clin Orthop Relat Res. 2017;475(9):2150-8.

20. Grosu I, Thienpont E, De Kock M, Scholtes JL, Lavand'homme P. Dynamic view of postoperative pain evolution after total knee arthroplasty: a prospective observational study. Minerva Anestesiol. 2016;82(3):274-83.

21. Hardy A, Sandiford MH, Menigaux C, Bauer T, Klouche S, Hardy P. Pain catastrophizing and pre-operative psychological state are predictive of chronic pain after joint arthroplasty of the hip, knee or shoulder: results of a prospective, comparative study at one year follow-up. Int Orthop. 2022;46(11):2461-9.

22. Heath EL, Ackerman IN, Cashman K, Lorimer M, Graves SE, Harris IA. Patient-reported outcomes after hip and knee arthroplasty : results from a large national registry. Bone Jt Open. 2021;2(6):422-32.

23. Jones CA, Voaklander DC, Johnston DW, Suarez-Almazor ME. Health related quality of life outcomes after total hip and knee arthroplasties in a community based population. J Rheumatol. 2000;27(7):1745-52.

24. Khalid S, Mohammad HR, Gooberman-Hill R, Garriga C, Pinedo-Villanueva R, Arden N, Price A, Wylde V, Peters TJ, Blom A, Judge A. Post-operative determinants of chronic pain after primary knee replacement surgery: Analysis of data on 258,386 patients from the National Joint Registry for England, Wales, Northern Ireland and the Isle of Man (NJR). Osteoarthr Cartil Open. 2021;3(1):100139.

25. Kim SH, Yoon KB, Yoon DM, Yoo JH, Ahn KR. Influence of Centrally Mediated Symptoms on Postoperative Pain in Osteoarthritis Patients Undergoing Total Knee Arthroplasty: A Prospective Observational Evaluation. Pain Pract. 2015;15(6):E46-53.

26. Kiran A, Bottomley N, Biant LC, Javaid MK, Carr AJ, Cooper C, Field RE, Murray DW, Price A, Beard DJ, Arden NK. Variations In Good Patient Reported Outcomes After Total Knee Arthroplasty. J Arthroplasty. 2015;30(8):1364-71.

27. Kornilov N, Lindberg MF, Gay C, Saraev A, Kuliaba T, Rosseland LA, Lerdal A. Higher physical activity and lower pain levels before surgery predict non-improvement of knee pain 1 year after TKA. Knee Surg Sports Traumatol Arthrosc. 2018;26(6):1698-708.

28. Kurien T, Arendt-Nielsen L, Petersen KK, Graven-Nielsen T, Scammell BE. Preoperative Neuropathic Pain-like Symptoms and Central Pain Mechanisms in Knee Osteoarthritis Predicts Poor Outcome 6 Months After Total Knee Replacement Surgery. J Pain. 2018;19(11):1329-41.

29. Larsen DB, Laursen M, Edwards RR, Simonsen O, Arendt-Nielsen L, Petersen KK. The Combination of Preoperative Pain, Conditioned Pain Modulation, and Pain Catastrophizing Predicts Postoperative Pain 12 Months After Total Knee Arthroplasty. Pain Med. 2021;22(7):1583-90.

30. Latijnhouwers D, Martini CH, Nelissen R, Verdegaal SHM, Vliet Vlieland TPM, Gademan MGJ, Longitudinal Leiden Orthopaedics Outcomes of Osteoarthritis Study G. Acute pain after total hip and knee arthroplasty does not affect chronic pain during the first postoperative year: observational cohort study of 389 patients. Rheumatol Int. 2022;42(4):689-98.

31. Lavand'homme PM, Grosu I, France MN, Thienpont E. Pain trajectories identify patients at risk of persistent pain after knee arthroplasty: an observational study. Clin Orthop Relat Res. 2014;472(5):1409-15.

32. Lee NK, Won SJ, Lee JY, Kang SB, Yoo SY, Chang CB. Presence of Night Pain, Neuropathic Pain, or Depressive Disorder Does Not Adversely Affect Outcomes After Total Knee Arthroplasty: A Prospective Cohort Study. J Korean Med Sci. 2022;37(43):e309.

33. Leppanen S, Niemelainen M, Huhtala H, Eskelinen A. Mild knee osteoarthritis predicts dissatisfaction after total knee arthroplasty: a prospective study of 186 patients aged 65 years or less with 2-year follow-up. BMC Musculoskelet Disord. 2021;22(1):657.

34. Leung YY, Lim Z, Fan Q, Wylde V, Xiong S, Yeo SJ, Lo NN, Chong HC, Yeo W, Tan MH, Chakraborty B, Bak-Siew Wong S, Thumboo J. Pre-operative pressure pain thresholds do not meaningfully explain satisfaction or improvement in pain after knee replacement: a cohort study. Osteoarthritis Cartilage. 2019;27(1):49-58.

35. Lundblad H, Kreicbergs A, Jansson KA. Prediction of persistent pain after total knee replacement for osteoarthritis. J Bone Joint Surg Br. 2008;90(2):166-71.

36. Lyman S, Lee YY, McLawhorn AS, Islam W, MacLean CH. What Are the Minimal and Substantial Improvements in the HOOS and KOOS and JR Versions After Total Joint Replacement? Clin Orthop Relat Res. 2018;476(12):2432-41.

37. Mahdi A, Halleberg-Nyman M, Wretenberg P. Preoperative psychological distress no reason to delay total knee arthroplasty: a register-based prospective cohort study of 458 patients. Arch Orthop Trauma Surg. 2020;140(11):1809-18.

38. Mekkawy KL, Zhang B, Wenzel A, Harris AB, Khanuja HS, Sterling RS, Hegde V, Oni JK. Mapping the course to recovery: a prospective study on the anatomic distribution of early postoperative pain after total knee arthroplasty. Arthroplasty. 2023;5(1):37.

39. Mercurio M, Gasparini G, Carbone EA, Galasso O, Segura-Garcia C. Personality traits predict residual pain after total hip and knee arthroplasty. Int Orthop. 2020;44(7):1263-70.

40. Mezey GA, Paulik E, Mate Z. Effect of osteoarthritis and its surgical treatment on patients' quality of life: a longitudinal study. BMC Musculoskelet Disord. 2023;24(1):537.

41. Musbahi O, Collins JE, Yang H, Selzer F, Chen AF, Lange J, Losina E, Katz JN. Assessment of Residual Pain and Dissatisfaction in Total Knee Arthroplasty: Methods Matter. JB JS Open Access. 2023;8(4):e23.00077.

42. Nishimoto J, Tanaka S, Inoue Y, Tanaka R. Minimal clinically important differences in short-term postoperative Knee injury and Osteoarthritis Outcome Score (KOOS) after total knee arthroplasty: A prospective cohort study. Journal of Orthopaedics, Trauma and Rehabilitation. 2023.

43. Noiseux NO, Callaghan JJ, Clark CR, Zimmerman MB, Sluka KA, Rakel BA. Preoperative predictors of pain following total knee arthroplasty. J Arthroplasty. 2014;29(7):1383-7.

44. Orr MN, Klika AK, Emara AK, Piuzzi NS, Cleveland Clinic Arthroplasty G. Combinations of Preoperative Patient-Reported Outcome Measure Phenotype (Pain, Function, and Mental Health) Predict Outcome After Total Knee Arthroplasty. J Arthroplasty. 2022;37(6S):S110-S20 e5.

45. Petersen KK, Arendt-Nielsen L, Simonsen O, Wilder-Smith O, Laursen MB. Presurgical assessment of temporal summation of pain predicts the development of chronic postoperative pain 12 months after total knee replacement. Pain. 2015;156(1):55-61.

46. Petersen KK, Simonsen O, Laursen MB, Arendt-Nielsen L. The Role of Preoperative Radiologic Severity, Sensory Testing, and Temporal Summation on Chronic Postoperative Pain Following Total Knee Arthroplasty. Clin J Pain. 2018;34(3):193-7.

47. Phillips JR, Hopwood B, Arthur C, Stroud R, Toms AD. The natural history of pain and neuropathic pain after knee replacement: a prospective cohort study of the point prevalence of pain and neuropathic pain to a minimum three-year follow-up. Bone Joint J. 2014;96-B(9):1227-33.

48. Priol R, Pasquier G, Putman S, Migaud H, Dartus J, Wattier JM. Trajectory of chronic and neuropathic pain, anxiety and depressive symptoms and pain catastrophizing after total knee replacement. Results of a prospective, single-center study at a mean follow-up of 7.5 years. Orthop Traumatol Surg Res. 2023;109(5):103543.

49. Pua YH, Poon CL, Seah FJ, Thumboo J, Clark RA, Tan MH, Chong HC, Tan JW, Chew ES, Yeo SJ. Predicting individual knee range of motion, knee pain, and walking limitation outcomes following total knee arthroplasty. Acta Orthop. 2019;90(2):179-86.

50. Quintana JM, Escobar A, Arostegui I, Bilbao A, Azkarate J, Goenaga JI, Arenaza JC. Health-related quality of life and appropriateness of knee or hip joint replacement. Arch Intern Med. 2006;166(2):220-6.

51. Rice DA, Kluger MT, McNair PJ, Lewis GN, Somogyi AA, Borotkanics R, Barratt DT, Walker M. Persistent postoperative pain after total knee arthroplasty: a prospective cohort study of potential risk factors. Br J Anaesth. 2018;121(4):804-12.

52. Sideris A, Malahias MA, Birch G, Zhong H, Rotundo V, Like BJ, Otero M, Sculco PK, Kirksey M. Identification of biological risk factors for persistent postoperative pain after total knee arthroplasty. Reg Anesth Pain Med. 2022;47(3):161-6.

53. Singh JA, Lewallen DG. Are outcomes after total knee arthroplasty worsening over time? A time-trends study of activity limitation and pain outcomes. BMC Musculoskelet Disord. 2014;15:440.

54. Solberg LI, Ziegenfuss JY, Rivard RL, Norton CK, Whitebird RR, Elwyn G, Swiontkowski M. Is There Room for Individual Patient-Specified Preferences in the Patient-Reported Outcome Measurement Revolution? J Patient Cent Res Rev. 2023;10(4):210-8.

55. Stephens G, Nightingale P, Mylogiannakis P, Suokas A. Do early patient reported outcome measures post total knee arthroplasty predict poor outcomes (the early PROMPT study). Physiotherapy Practice and Research. 2020;41(2):109-20.

56. Tang S, Jin Y, Hou Y, Wang W, Zhang J, Zhu W, Zhang W, Gu X, Ma Z. Predictors of Chronic Pain in Elderly Patients Undergoing Total Knee and Hip Arthroplasty: A Prospective Observational Study. J Arthroplasty. 2023;38(9):1693-9.

57. Terradas-Monllor M, Ruiz MA, Ochandorena-Acha M. Postoperative Psychological Predictors for Chronic Postsurgical Pain After a Knee Arthroplasty: A Prospective Observational Study. Phys Ther. 2024;104(1).

58. Thomazeau J, Rouquette A, Martinez V, Rabuel C, Prince N, Laplanche JL, Nizard R, Bergmann JF, Perrot S, Lloret-Linares C. Predictive Factors of Chronic Post-Surgical Pain at 6 Months Following Knee Replacement: Influence of Postoperative Pain Trajectory and Genetics. Pain Physician. 2016;19(5):E729-41.

59. Tian M, Li Z, Chen X, Wu Q, Shi H, Zhu Y, Shi Y. Prevalence and Predictors of Chronic Pain with Two-Year Follow-Up After Knee Arthroplasty. J Pain Res. 2022;15:1091-105.

60. Utrillas-Compaired A, De la Torre-Escuredo BJ, Tebar-Martinez AJ, Asunsolo-Del Barco A. Does preoperative psychologic distress influence pain, function, and quality of life after TKA? Clin Orthop Relat Res. 2014;472(8):2457-65.

61. van der Wees PJ, Wammes JJ, Akkermans RP, Koetsenruijter J, Westert GP, van Kampen A, Hannink G, de Waal-Malefijt M, Schreurs BW. Patient-reported health outcomes after total hip and knee surgery in a Dutch University Hospital Setting: results of twenty years clinical registry. BMC Musculoskelet Disord. 2017;18(1):97.

62. Vina ER, Ran D, Ashbeck EL, Kwoh CK. Widespread Pain Is Associated with Increased Risk of No Clinical Improvement After TKA in Women. Clin Orthop Relat Res. 2020;478(7):1453.

63. Vuorenmaa M, Ylinen J, Kiviranta I, Intke A, Kautiainen HJ, Malkia E, Hakkinen A. Changes in pain and physical function during waiting time and 3 months after knee joint arthroplasty. J Rehabil Med. 2008;40(7):570-5.

64. W-Dahl A, Sundberg M, Lidgren L, Ranstam J, Robertsson O. An examination of the effect of different methods of scoring pain after a total knee replacement on the number of patients who report unchanged or worse pain. Bone Joint J. 2014;96-B(9):1222-6.

65. Waimann CA, Fernandez-Mazarambroz RJ, Cantor SB, Lopez-Olivo MA, Zhang H, Landon GC, Siff SJ, Suarez-Almazor ME. Cost-effectiveness of total knee replacement: a prospective cohort study. Arthritis Care Res (Hoboken). 2014;66(4):592-9.

66. Wylde V, Palmer S, Learmonth ID, Dieppe P. The association between pre-operative pain sensitisation and chronic pain after knee replacement: an exploratory study. Osteoarthritis Cartilage. 2013;21(9):1253-6.

67. Wylde V, Penfold C, Rose A, Blom AW. Variability in long-term pain and function trajectories after total knee replacement: A cohort study. Orthop Traumatol Surg Res. 2019;105(7):1345-50.

68. Yan Z, Liu M, Wang X, Wang J, Wang Z, Liu J, Wu S, Luan X. Construction and Validation of Machine Learning Algorithms to Predict Chronic Post-Surgical Pain Among Patients Undergoing Total Knee Arthroplasty. Pain Manag Nurs. 2023;24(6):627-33.

69. Buus AAO, Laugesen B, El-Galaly A, Laursen M, Hejlesen OK. The potential of dividing the oxford knee score into subscales for predicting clinically meaningful improvements in pain and function of patients undergoing total knee arthroplasty. Int J Orthop Trauma Nurs. 2022;45:100919.

70. Escobar A, García Pérez L, Herrera-Espiñeira C, Aizpuru F, Sarasqueta C, Gonzalez Sáenz de Tejada M, et al. Total knee replacement; minimal clinically important differences and responders. Osteoarthritis Cartilage. 2013;21(12):2006-12.

71. Berliner JL, Brodke DJ, Chan V, SooHoo NF, Bozic KJ. Can Preoperative Patient-reported Outcome Measures Be Used to Predict Meaningful Improvement in Function After TKA? Clin Orthop Relat Res. 2017;475(1):

72. Escobar A, Riddle DL. Concordance between important change and acceptable symptom state following knee arthroplasty: the role of baseline scores. Osteoarthritis Cartilage. 2014;22(8):1107-10. 149-57.

## S3.1 Mean age and range


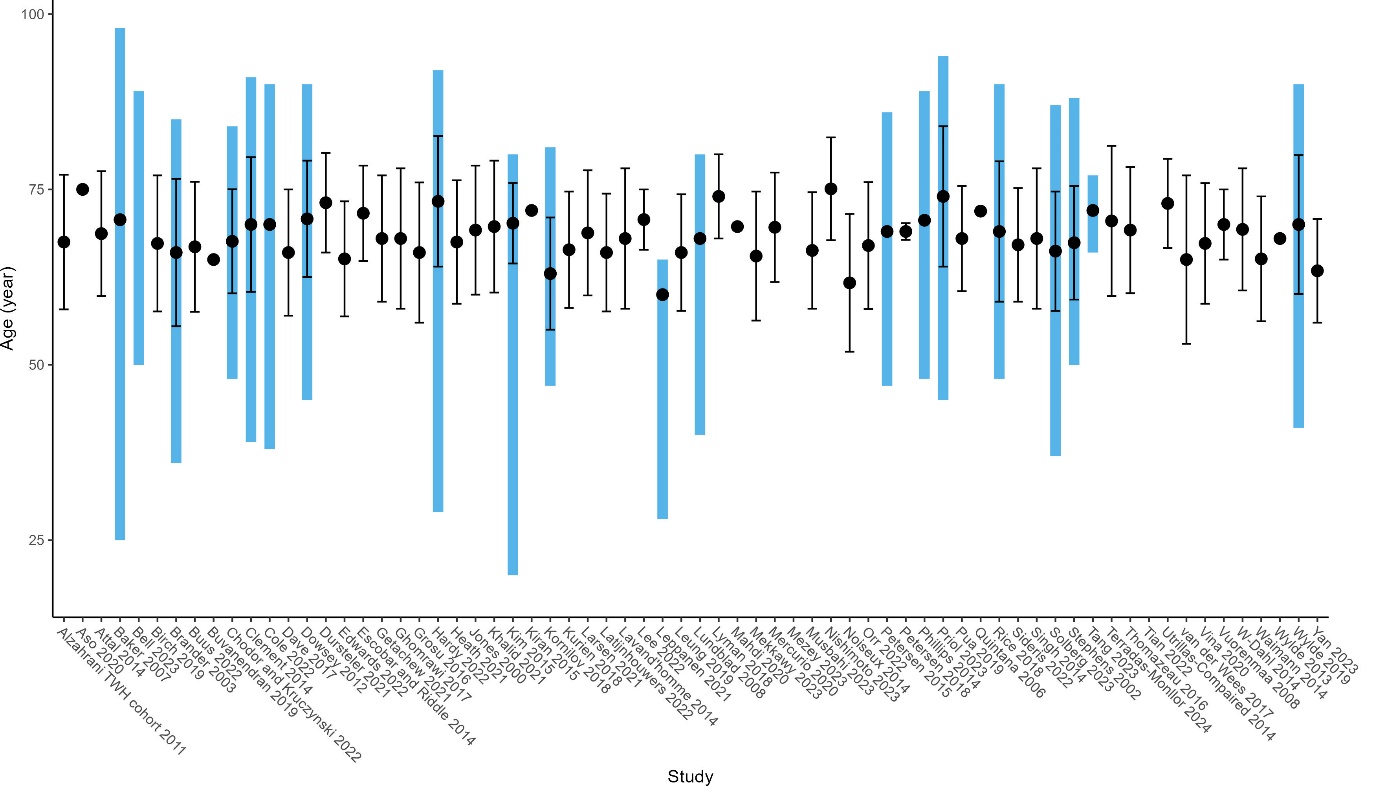


Figure S3.1. Mean age and their standard deviations reported in the individual studies. Range of age was plotted as blue bars.

## S3.2 Proportion of females


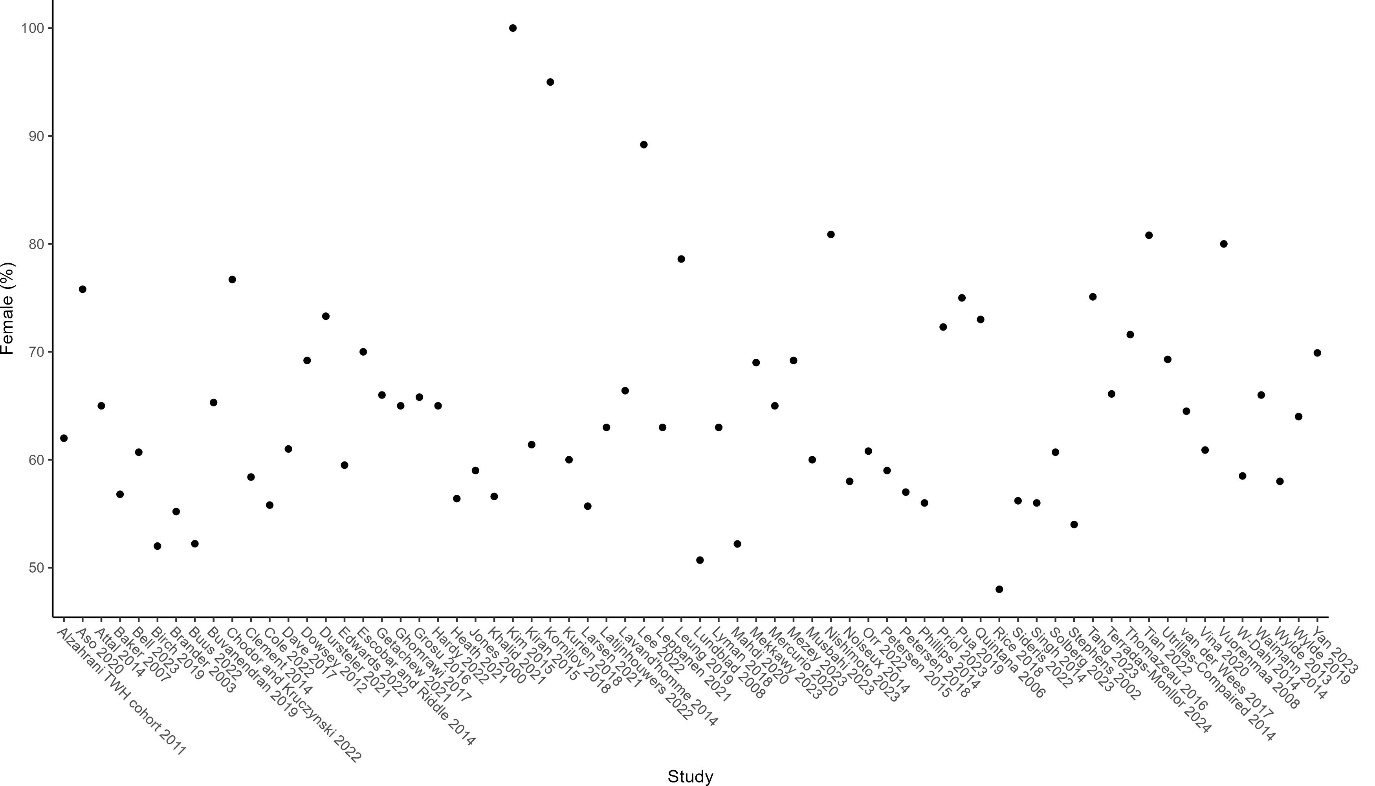


Figure S3.2. Proportion of females reported in the individual studies

## S3.3 Data collection timeframe


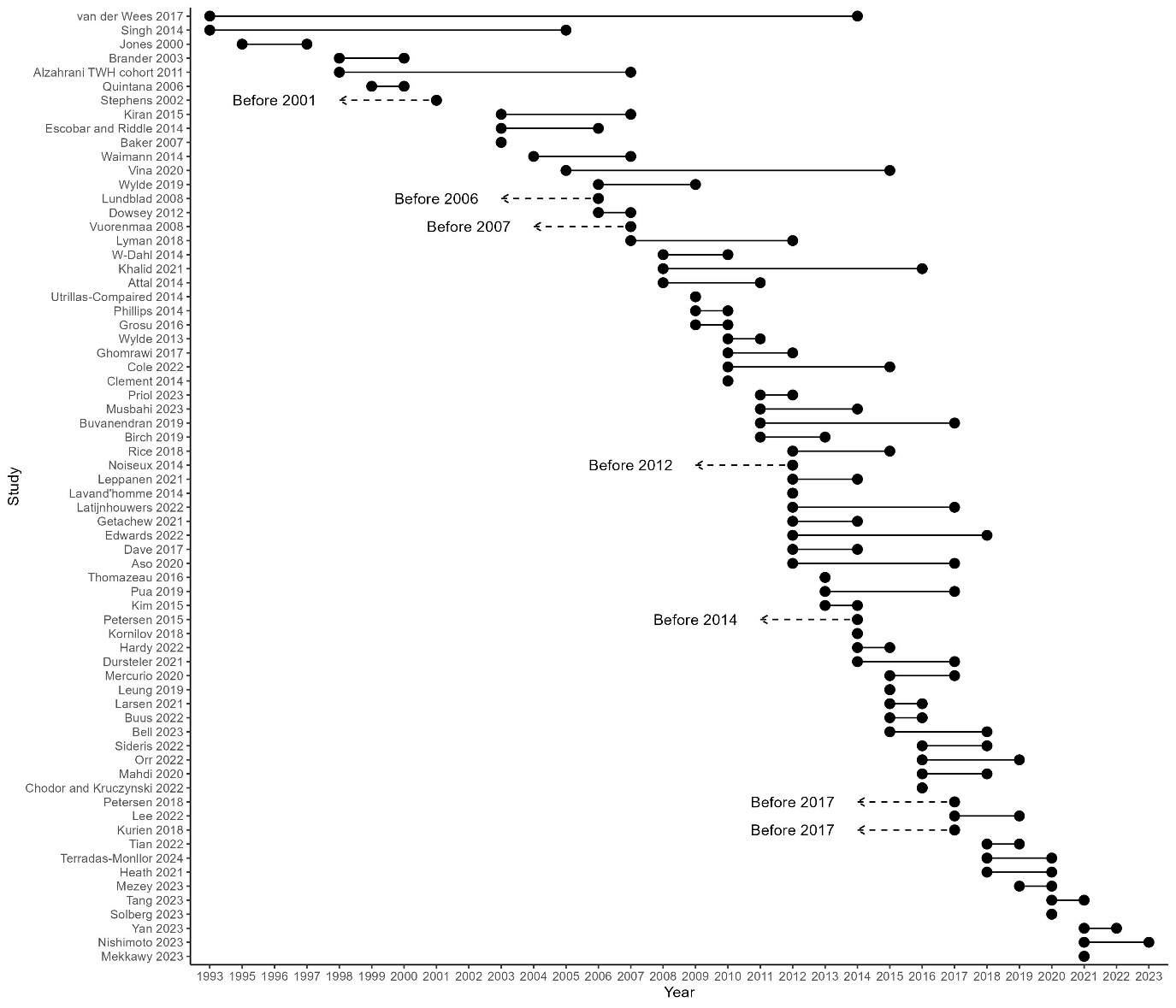


Figure S3.3. Data collection timeframe in the individual studies.

## S3.4 Proportions of lost to follow-ups and revisions


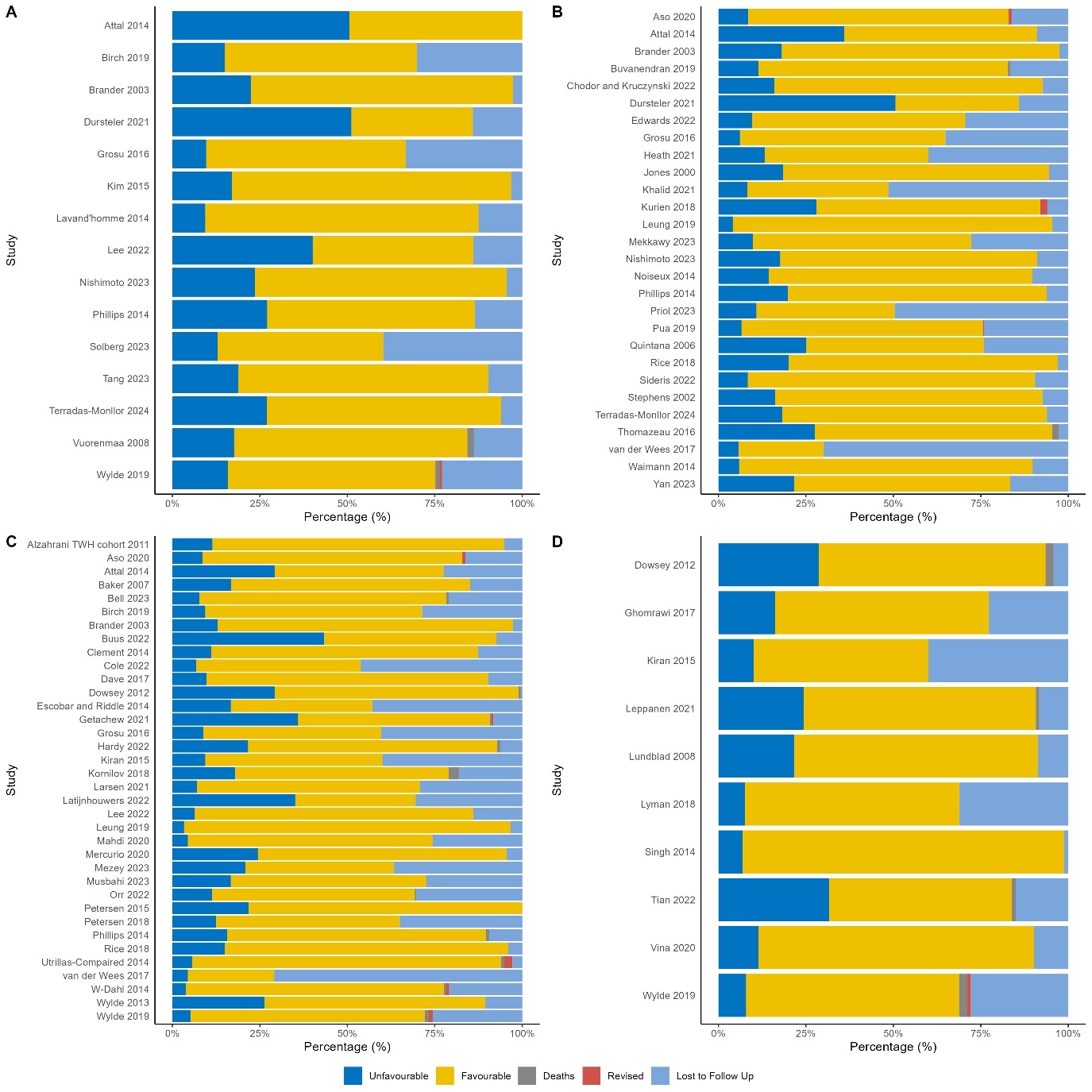


Figure S3.4. Favourable and unfavourable pain outcomes and reasons of missing data reported in 3, 6, 12, and 24 months (represented in sub-plots A, B, C, and D, respectively) in TKR studies.

# S4. Traffic light plot of the risk of bias assessments in TKR studies

The corresponding domains in the figures are:

- D1: Was the study's target population a close representation of the national population in relation to relevant variables?
- D2: Was the sampling frame a true or close representation of the target population?
- D3: Was some form of random selection used to select the sample, OR was a census undertaken?
- D4: Was the likelihood of nonresponse bias minimal?
- D5: Were data collected directly from the subjects (as opposed to a proxy)?
- D6: Was an acceptable case definition used in the study?
- D7: Was the study instrument that measured the parameter of interest shown to have validity and reliability?
- D8: Was the same mode of data collection used for all subjects?
- D9: Was the length of the shortest prevalence period for the parameter of interest appropriate?
- D10: Were the numerator(s) and denominator(s) for the parameter of interest appropriate?

## S4.1 TKR studies (3 months)


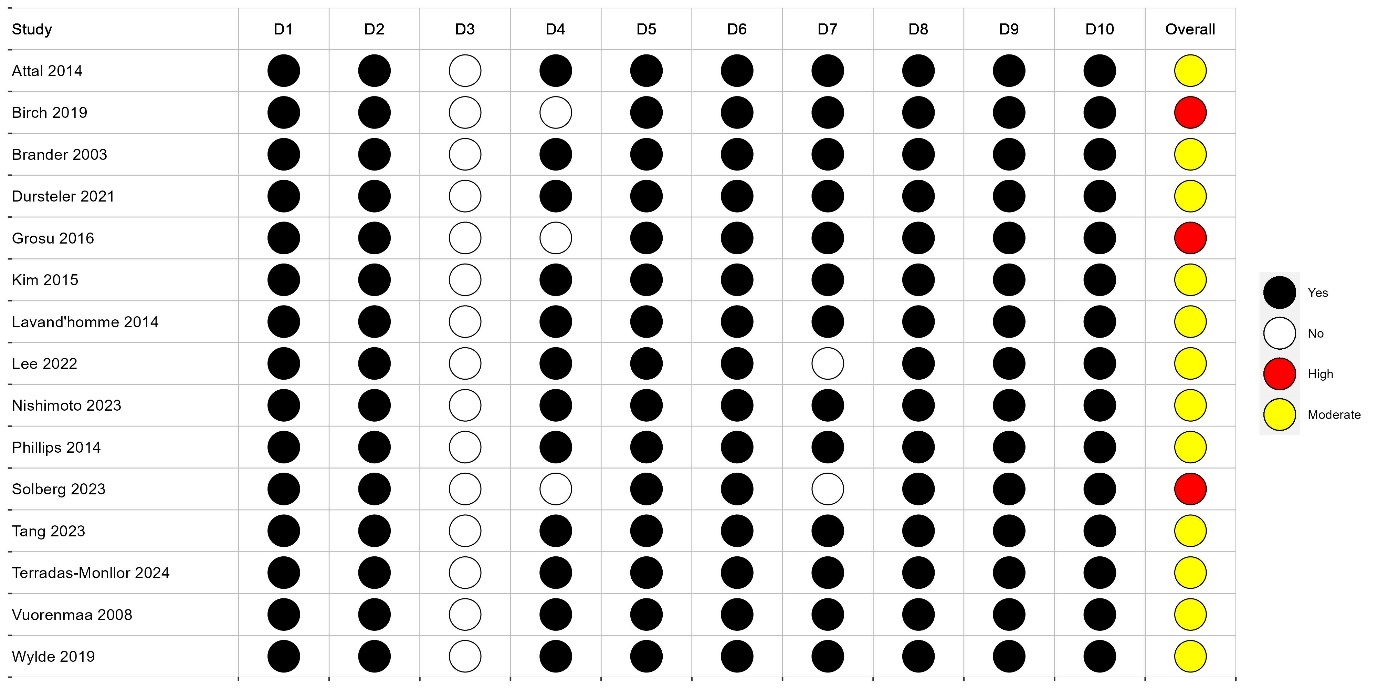


## S4.2 TKR studies (6 months)


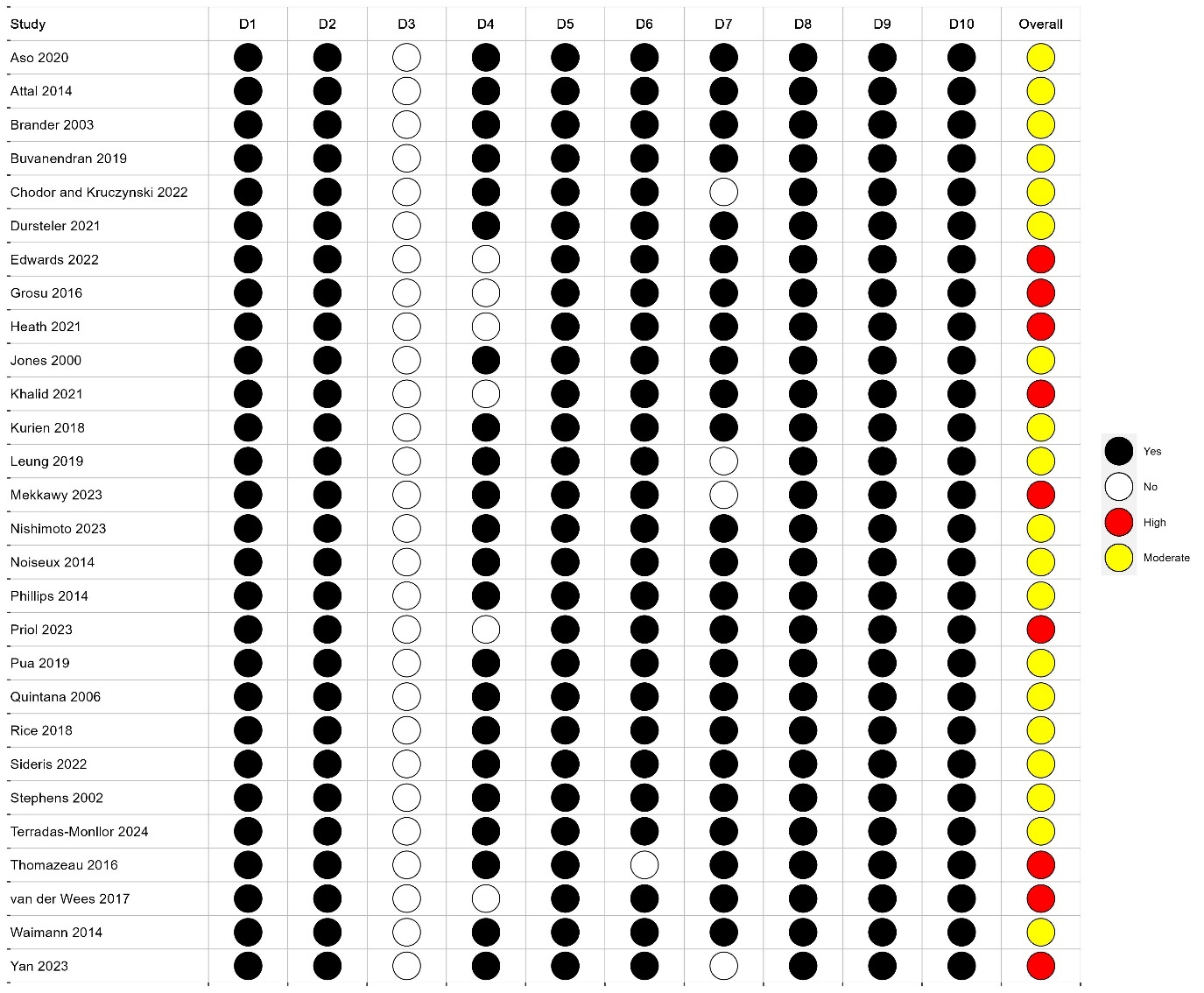


## S4.3 TKR studies (12 months)


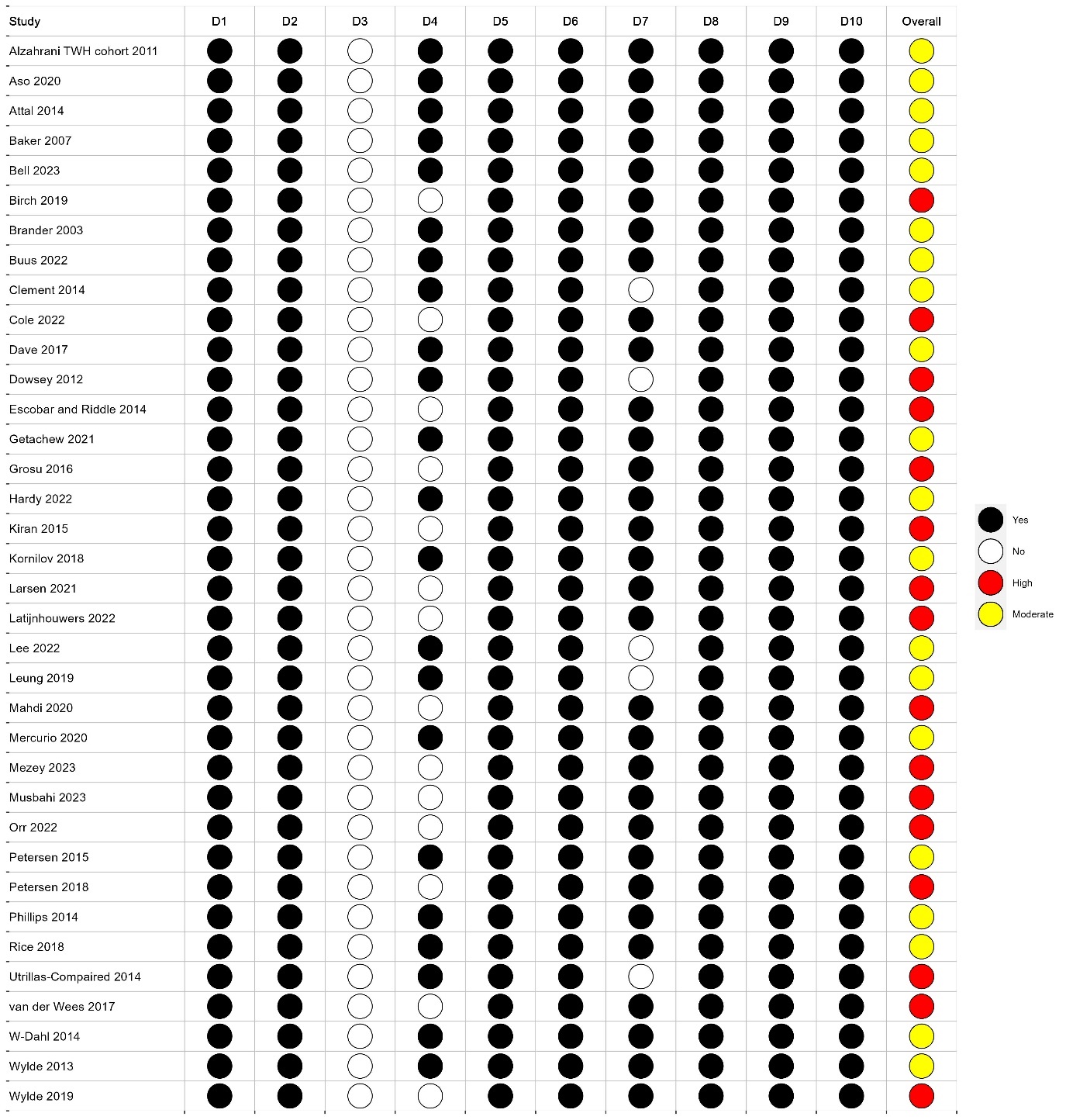


## S4.4 TKR studies (24 months)


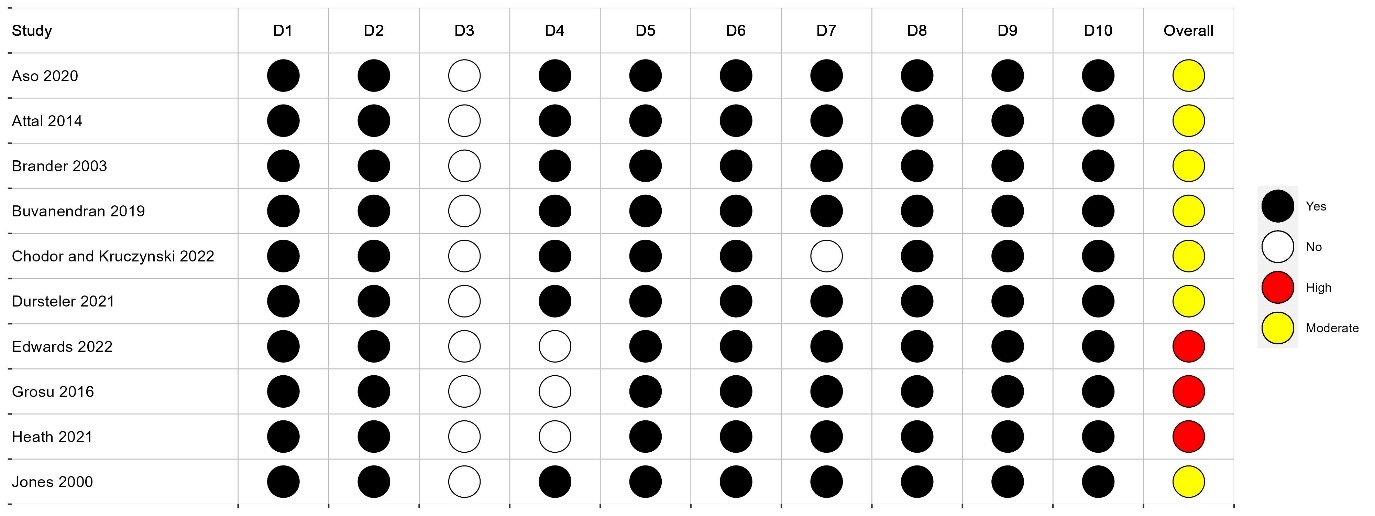


# S5. Forest plots of univariate meta-analyses in TKR studies

## S5.1 TKR studies (3 months)


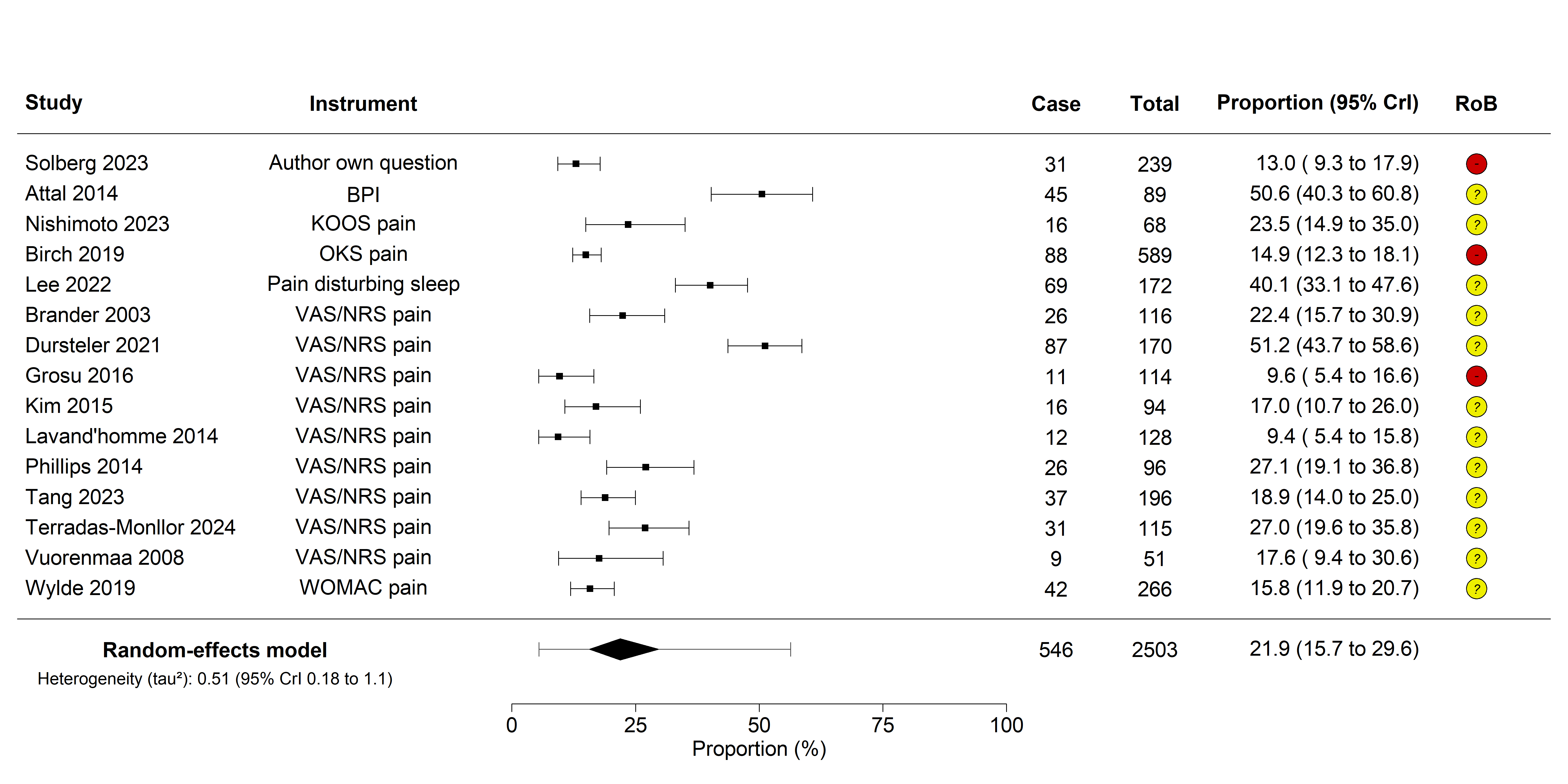


## S5.2 TKR studies (6 months)


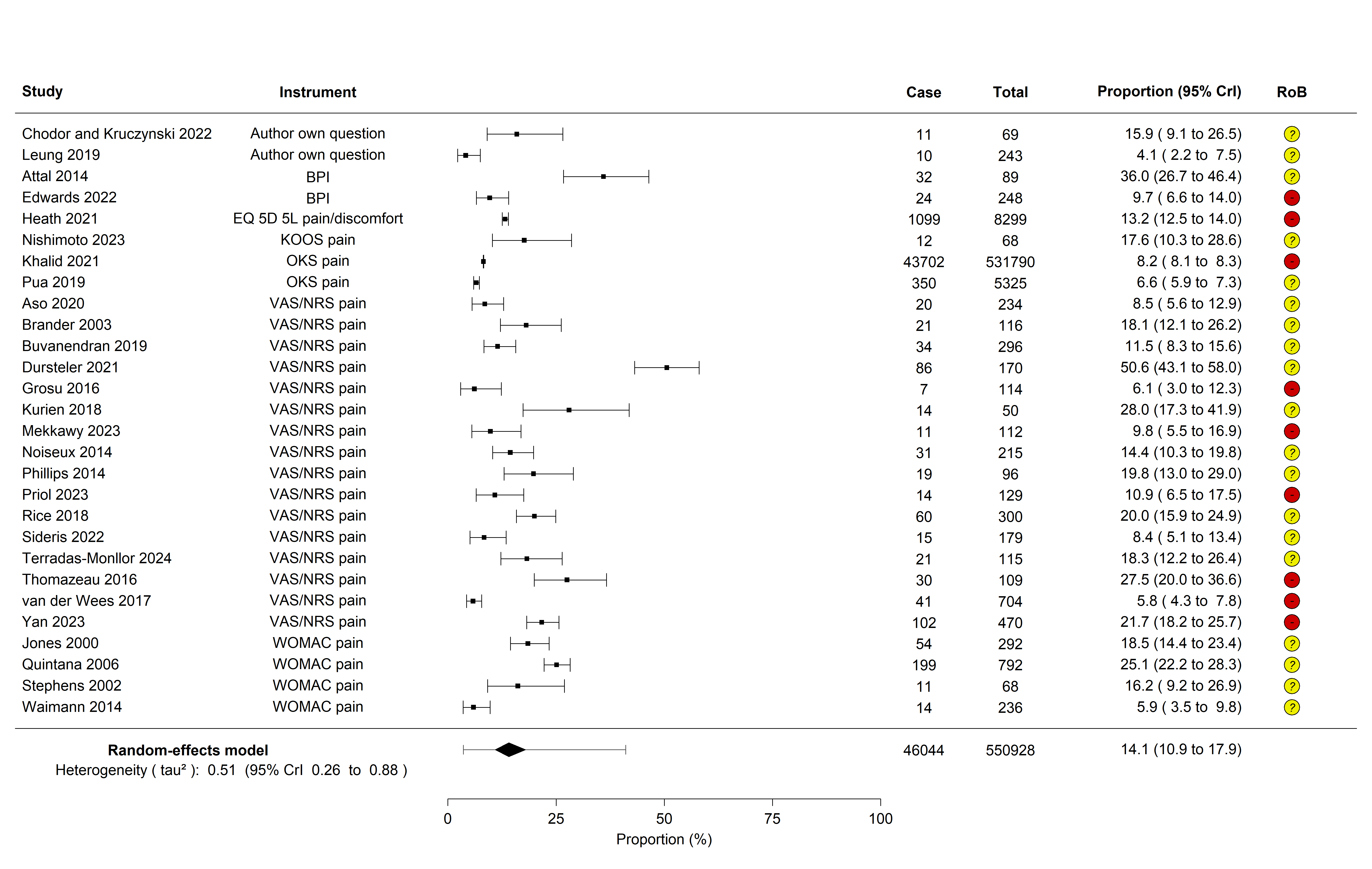


## S5.3 TKR studies (12 months)


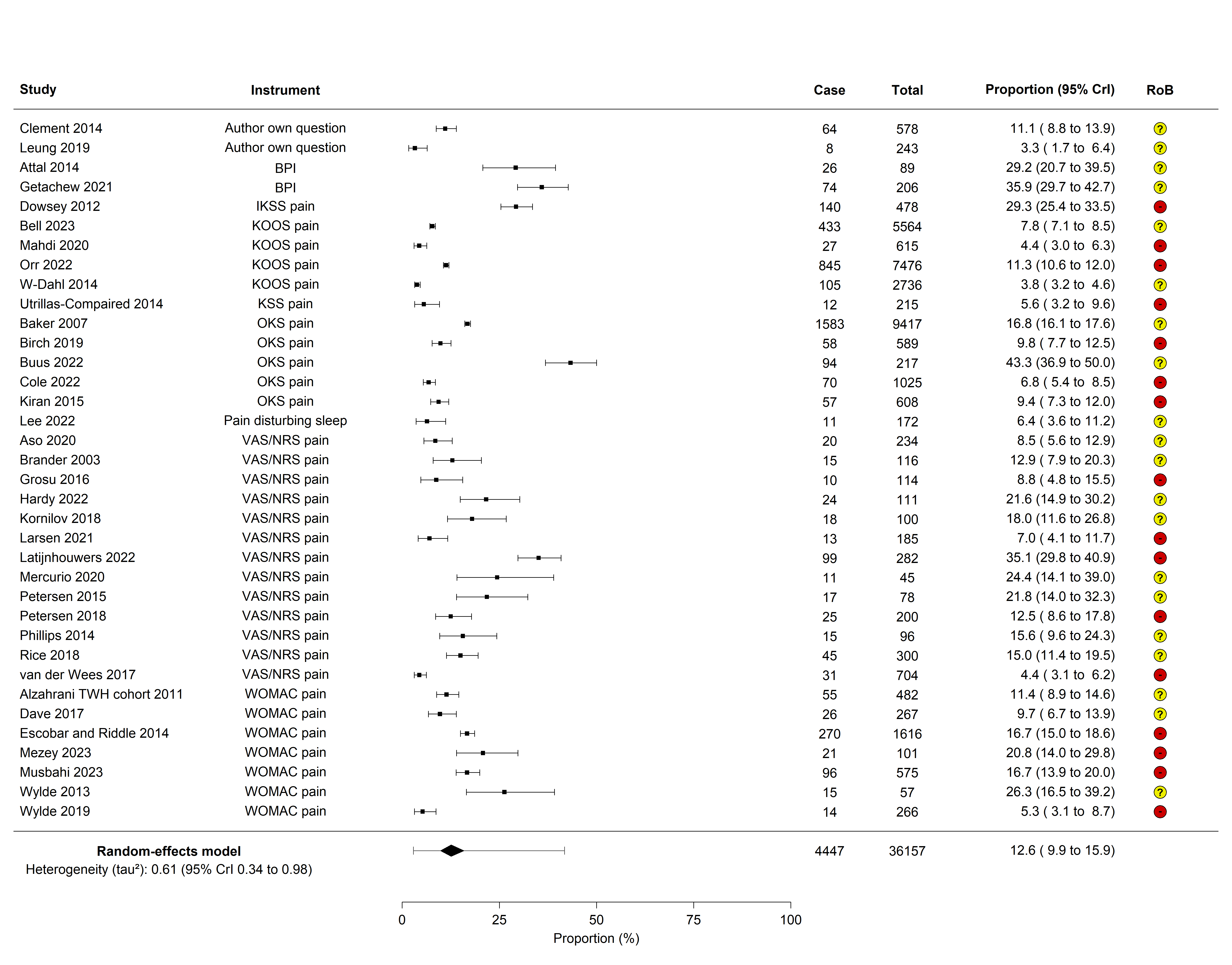


## S5.4 TKR studies (24 months)


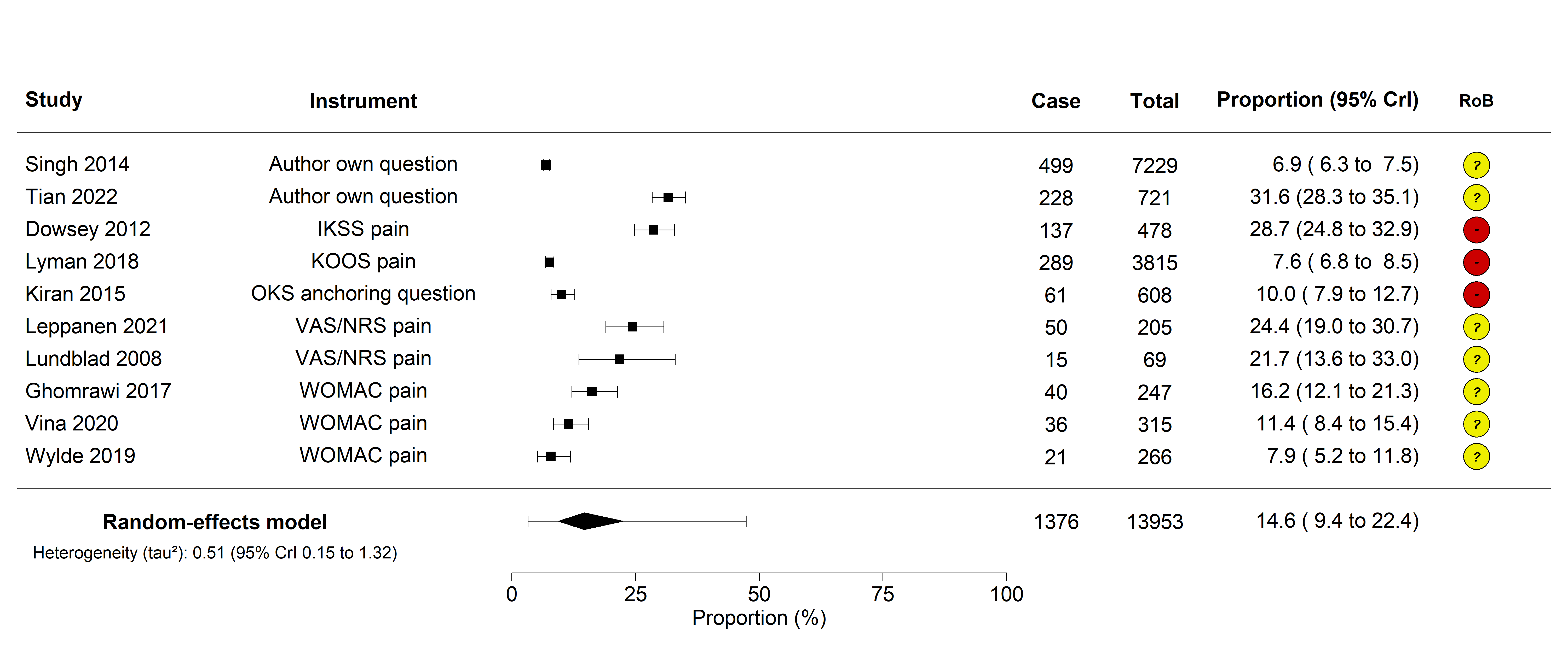


# S6. Table of multivariate and univariate meta-analysis results in TKR studies

|  | Multivariate meta-analysis | | Univariate meta-analysis | |
| --- | --- | --- | --- | --- |
| Time | Median (95% CrI) | tau² (95% CrI) | Median (95% CrI) | tau² (95% CrI) |
| 3 months | 21.2  (16.9 to 26.4) | 0.49  (0.28 to 0.91) | 21.9  (15.6 to 29.4) | 0.51  (0.18 to 1.1) |
| 6 months | 14.6  (11.9 to 17.8) | 0.56  (0.34 to 0.91) | 14.1  (10.9 to 17.9) | 0.51  (0.27 to 0.9) |
| 12 months | 12.6  (10.3 to 15.5) | 0.63  (0.41 to 0.99) | 12.6  (9.9 to 15.9) | 0.61  (0.35 to 0.99) |
| 24 months | 14.2  (10 to 20.1) | 0.58  (0.25 to 1.55) | 14.6  (9.5 to 22.4) | 0.52  (0.16 to 1.35) |

# S7. Meta-regression analyses in TKR studies

## S7.1 Mean age

| Time | No. studies | slope | intercept |
| --- | --- | --- | --- |
| 3 months | 15 | 0.133 | -1.272 |
| 6 months | 28 | 0.082 | -1.851 |
| 12 months | 34 | -0.029 | -1.942 |
| 24 months | 9 | -0.073 | -1.886 |

## S7.2 Proportion of females

| Time | No. studies | slope | intercept |
| --- | --- | --- | --- |
| 3 months | 15 | 0.009 | -1.273 |
| 6 months | 28 | -0.040 | -1.697 |
| 12 months | 36 | -0.006 | -1.939 |
| 24 months | 10 | 0.045 | -1.798 |

## S7.3 Sample sizes

| Time | No. studies | slope | intercept |
| --- | --- | --- | --- |
| 3 months | 15 | -0.001 | -1.269 |
| 6 months | 28 | 0.000 | -1.785 |
| 12 months | 36 | 0.000 | -1.936 |
| 24 months | 10 | 0.000 | -1.750 |

# S8. Subgroup analyses in TKR studies

• Geographic region (categorical; North America, Asia, Europe, and Australia)

• Data source (categorical; surgeons, single hospital, multi-centre, and national registry

• Pain outcomes instruments (categorical; multidimensional, e.g. WOMAC pain, simple, e.g. VAS/NRS and EQ-5D 5L, and not validated, e.g. author’s own questionnaires)

• Cut-off definitions (categorical; based on MCID, based on PASS, based on pain intensity, e.g. specific post-operative VAS values, based on functional impact, e.g. night pain, pain on movement, or limiting daily life, based on symptom improvement, e.g. no change or increase in pain from pre-operative)

## S8.1 Geographical regions

| Subgroup | No. Studies | Median (95% CrI) | tau² (95% CrI) |
| --- | --- | --- | --- |
| 3 Months | | | |
| Asia | 4 | 24.26 (11.85 to 42.3) | 0.32 (0 to 2.34) |
| Europe | 9 | 22.17 (12.42 to 35.18) | 0.77 (0.19 to 2.17) |
| North America | 2 | 16.63 (0.92 to 81.83) | 0.21 (0 to 31.7) |
| 6 Months | | | |
| Asia | 5 | 9.91 (4.04 to 21.69) | 0.64 (0.06 to 3.19) |
| Australia | 2 | 15.53 (1.23 to 73.87) | 0.19 (0 to 23.85) |
| Europe | 12 | 17.99 (10.88 to 27.3) | 0.77 (0.27 to 1.85) |
| North America | 9 | 11.87 (8.87 to 15.58) | 0.13 (0 to 0.45) |
| 12 Months | | | |
| Asia | 3 | 5.81 (2.2 to 12.88) | 0.12 (0 to 2.76) |
| Australia | 2 | 21.45 (0.1 to 98.64) | 0.73 (0 to 97.26) |
| Europe | 25 | 13.54 (9.91 to 18.16) | 0.72 (0.37 to 1.29) |
| North America | 6 | 11.15 (8.31 to 14.9) | 0.09 (0.01 to 0.4) |
| 24 Months | | | |
| Asia | 1 | 31.36 (28.02 to 34.79) | NA |
| Australia | 1 | 28.29 (24.49 to 32.56) | NA |
| Europe | 4 | 14.29 (5.86 to 32.4) | 0.47 (0.03 to 3.51) |
| North America | 4 | 9.56 (5.19 to 17.04) | 0.2 (0 to 1.45) |

## S8.2 Setting

| Subgroup | No. Studies | Median (95% CrI) | tau² (95% CrI) |
| --- | --- | --- | --- |
| 3 Months | | | |
| Other | 1 | 26.44 (19.1 to 34.38) | NA |
| Single hospital | 8 | 25.41 (15.64 to 38.72) | 0.55 (0.13 to 1.73) |
| Surgeon | 6 | 16.89 (8.43 to 29.76) | 0.55 (0.07 to 2.2) |
| 6 Months | | | |
| Multicentre | 6 | 13.84 (7.93 to 22.5) | 0.35 (0.04 to 1.41) |
| Other | 2 | 18.34 (7.93 to 37.29) | 0.02 (0 to 2.68) |
| Registry | 1 | 8.22 (8.14 to 8.29) | NA |
| Single hospital | 16 | 15.03 (9.79 to 21.64) | 0.73 (0.3 to 1.55) |
| Surgeon | 3 | 10.82 (3.35 to 30.39) | 0.26 (0 to 5.13) |
| 12 Months | | | |
| Multicentre | 12 | 11.29 (7.37 to 16.83) | 0.56 (0.19 to 1.31) |
| Registry | 1 | 16.80 (16.07 to 17.57) | NA |
| Single hospital | 20 | 13.96 (9.77 to 19.93) | 0.77 (0.34 to 1.47) |
| Surgeon | 3 | 8.93 (4.2 to 16.32) | 0.05 (0 to 1.6) |
| 24 Months | | | |
| Multicentre | 1 | 11.50 (8.14 to 15.03) | NA |
| Single hospital | 9 | 14.98 (9.11 to 23.7) | 0.57 (0.16 to 1.54) |

## S8.3 Pain outcome instruments

| Subgroup | No. Studies | Median (95% CrI) | tau² (95% CrI) |
| --- | --- | --- | --- |
| 3 Months | | | |
| Multidimensional | 5 | 26.76 (12.16 to 49.19) | 0.7 (0.09 to 3.37) |
| Not validated | 1 | 12.98 (9.3 to 17.9) | NA |
| Simple | 9 | 20.6 (13.08 to 31.41) | 0.5 (0.11 to 1.47) |
| 6 Months | | | |
| Multidimensional | 9 | 13.68 (8.49 to 22.5) | 0.56 (0.14 to 1.57) |
| Not validated | 2 | 7.65 (0 to 99.79) | 1.72 (0 to 219.88) |
| Simple | 17 | 15.15 (10.99 to 20.57) | 0.49 (0.2 to 1.03) |
| 12 Months | | | |
| Multidimensional | 21 | 12.67 (8.95 to 17.66) | 0.72 (0.33 to 1.34) |
| Not validated | 2 | 6.51 (0 to 98.5) | 1.52 (0 to 166.77) |
| Simple | 13 | 13.91 (9.63 to 19.73) | 0.44 (0.14 to 1.02) |
| 24 Months | | | |
| Multidimensional | 6 | 12.39 (6.86 to 20.55) | 0.41 (0.07 to 1.58) |
| Not validated | 2 | 15.65 (0 to 99.99) | 3.63 (0.07 to 399.6) |
| Simple | 2 | 23.53 (8.04 to 48) | 0.02 (0 to 4.48) |

## S8.4 Cut-off definitions

| Subgroup | No. Studies | Median (95% CrI) | tau² (95% CrI) |
| --- | --- | --- | --- |
| 3 Months | | | |
| Based on functional impact | 1 | 40.1 (33.1 to 47.6) | NA |
| Based on MCID | 2 | 17.91 (4.01 to 63.34) | NA |
| Based on pain intensity | 10 | 21.61 (13.37 to 33.15) | 0.66 (0.18 to 1.74) |
| Based on symptom improvement | 2 | 18.26 (0.26 to 95.83) | NA |
| 6 Months | | | |
| Based on functional impact | 1 | 15.9 (9.1 to 26.5) | NA |
| Based on MCID | 3 | 14.38 (1.88 to 56.47) | 1 (0.05 to 13.77) |
| Based on pain intensity | 21 | 15.26 (11.63 to 19.96) | 0.48 (0.23 to 0.92) |
| Based on symptom improvement | 3 | 6.75 (1.68 to 26.53) | 0.44 (0 to 7.44) |
| 12 Months | | | |
| Based on functional impact | 1 | 6.4 (3.6 to 11.2) | NA |
| Based on MCID | 5 | 15.77 (5.83 to 36.93) | 0.92 (0.13 to 4.38) |
| Based on pain intensity | 19 | 14.34 (10.16 to 19.75) | 0.61 (0.26 to 1.19) |
| Based on PASS | 2 | 13.7 (1.08 to 66.8) | NA |
| Based on symptom improvement | 9 | 8.86 (5.08 to 15.27) | 0.63 (0.15 to 1.81) |
| 24 Months | | | |
| Based on functional impact | 1 | 31.6 (28.3 to 35.1) | NA |
| Based on MCID | 3 | 10.88 (4.18 to 25.04) | 0.22 (0 to 3.32) |
| Based on pain intensity | 4 | 18.24 (6.02 to 43.02) | 0.75 (0.08 to 5.12) |
| Based on symptom improvement | 2 | 9.15 (2.73 to 24.14) | NA |

# S9. Doi plots and the LFK indexes in TKR studies

## S9.1 TKR studies (3 months)


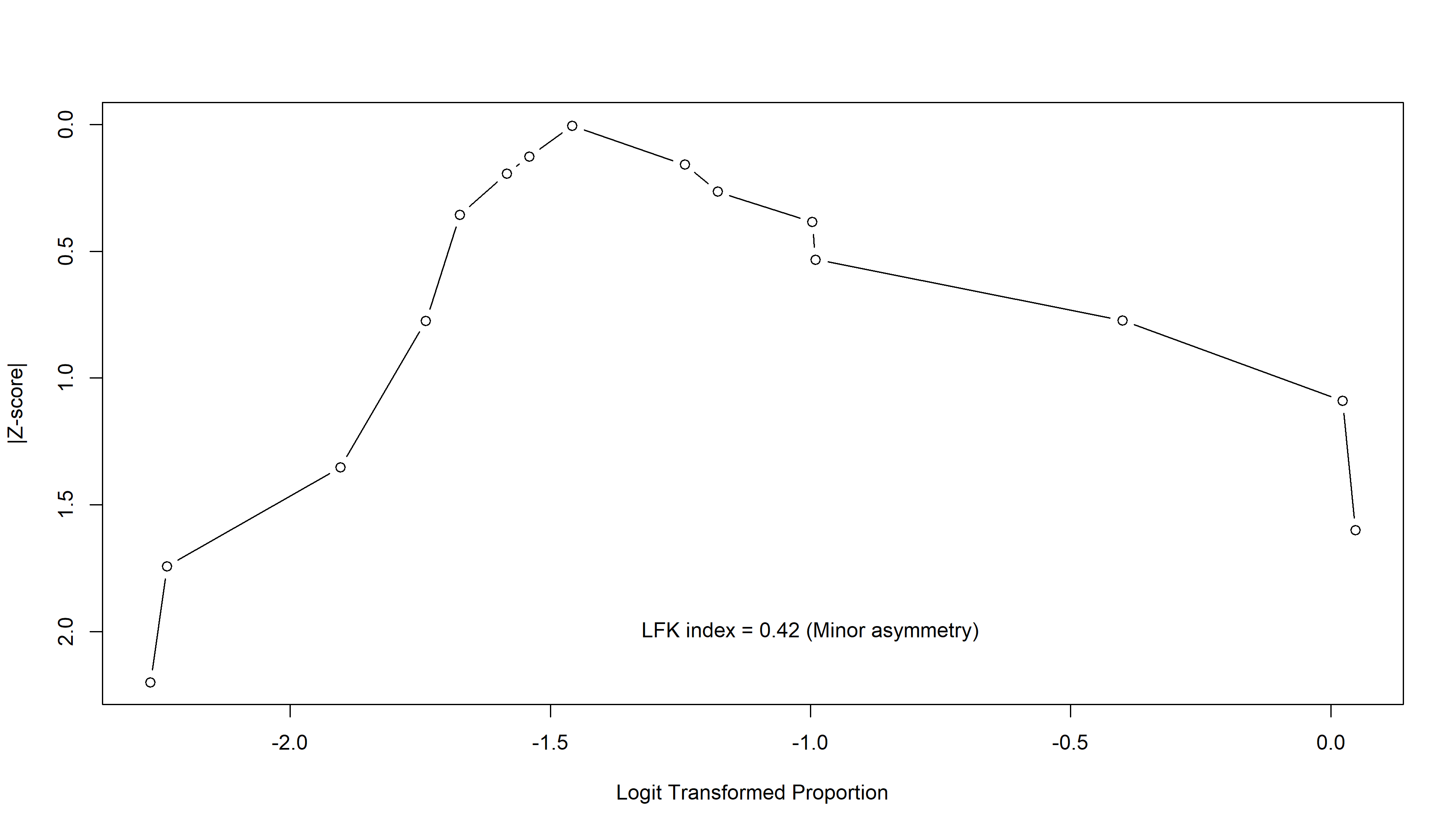


## S9.2 TKR studies (6 months)


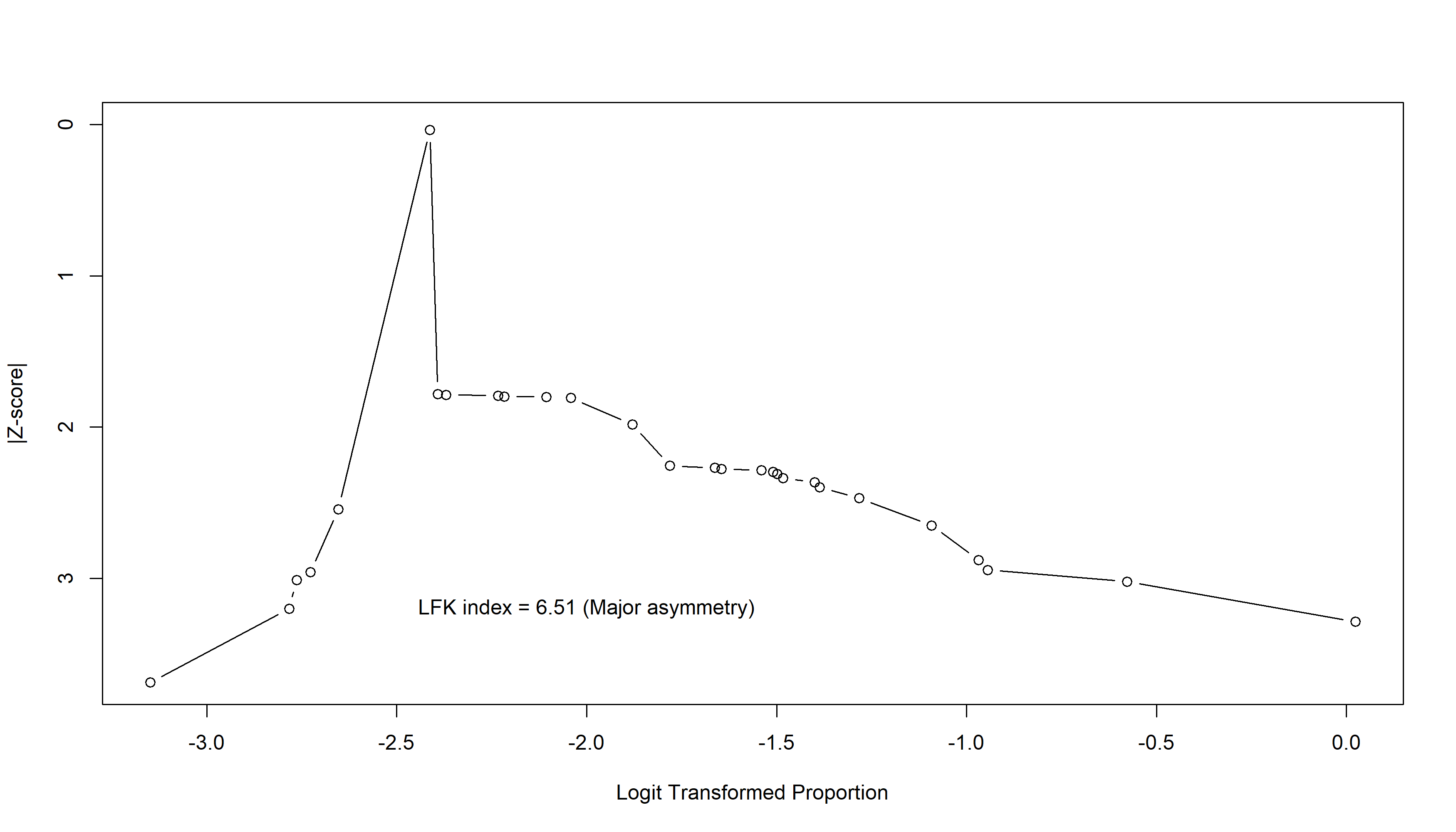


## S9.3 TKR studies (12 months)


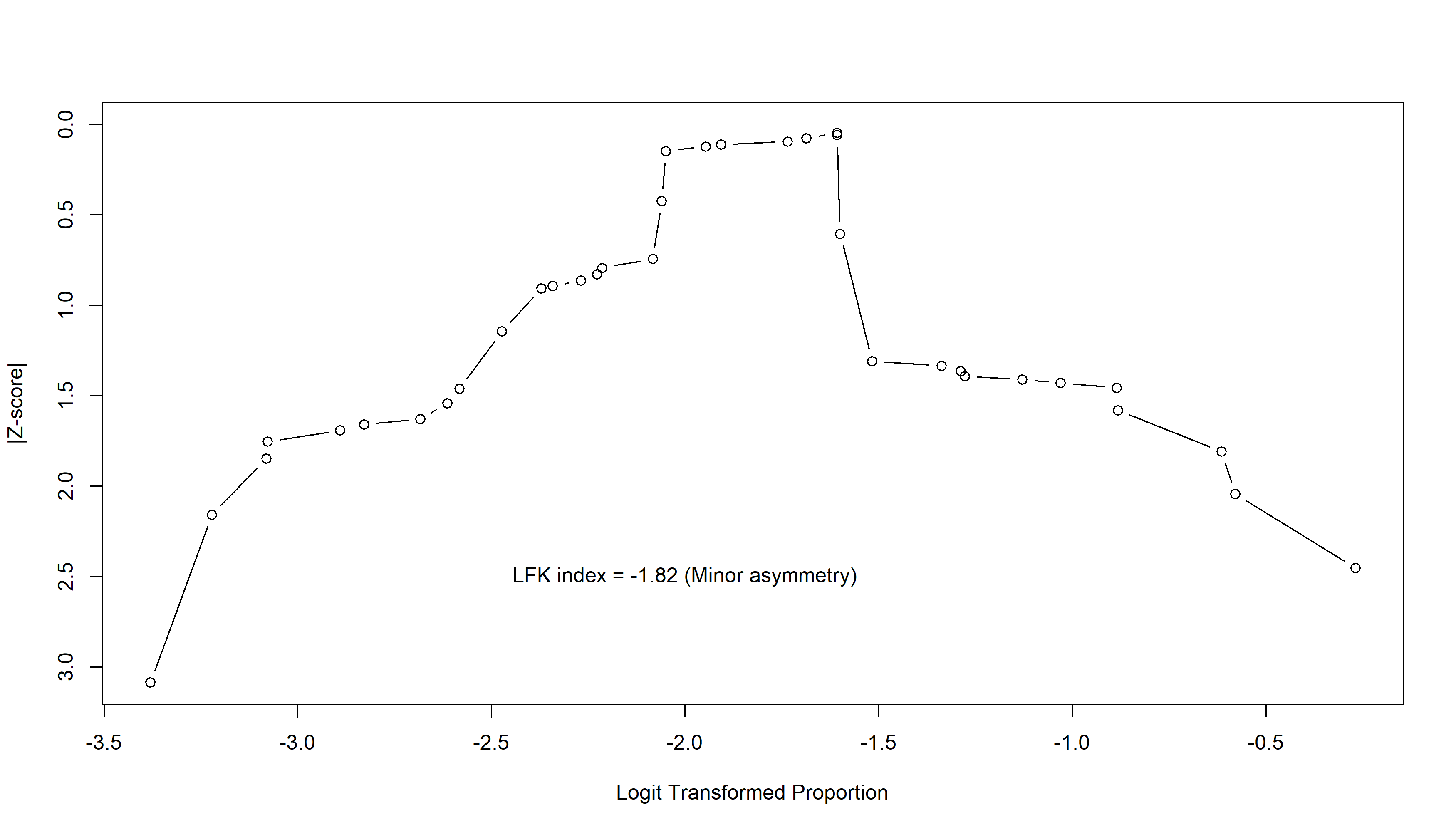


## S9.4 TKR studies (24 months)


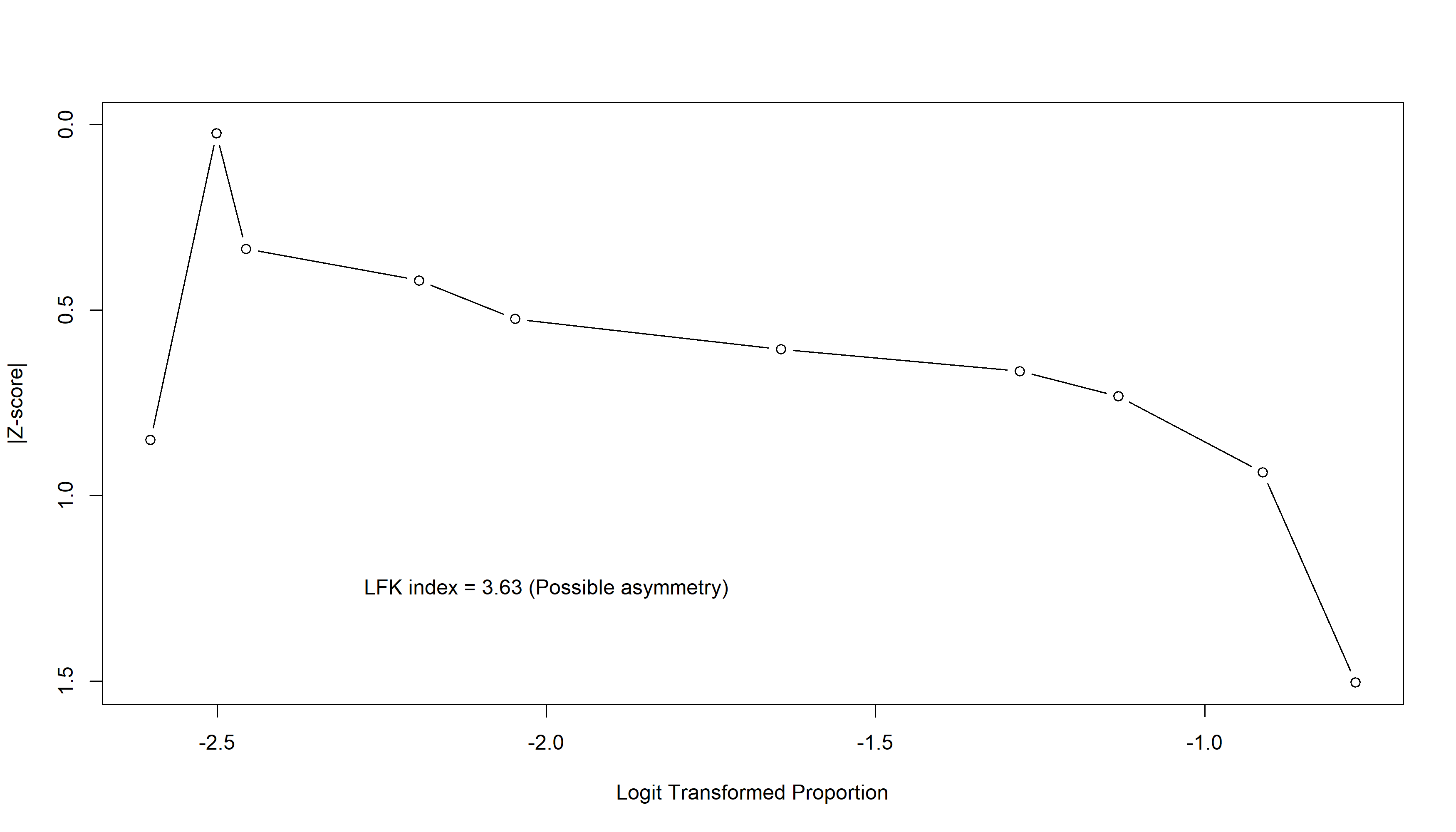


# S10. Sensitivity analyses

In the sensitivity analysis, we excluded the following studies based on their unique clinical characteristics:

- Tang 2023 (impact on 3 months results only)
- Leppanen 2021 (impact on 24 months results only)
- Fast track studies (impact on 3 and 12 months results only)
- Mekkawy 2023 and Yan 2023 (impact on 6 months results only)
- Studies on TKR or UKR operations
- Studies with more than 20% lost to follow-up
- High risk of bias studies

| Name | No. studies | Median (95% CrI) | tau² (95% CrI) |
| --- | --- | --- | --- |
| 3 Months | | | |
| Excluding Tang 2023 | 14 | 22.12 (15.4 to 30.2) | 0.55 (0.19 to 1.21) |
| Excluding Fast track studies | 14 | 22.56 (15.96 to 30.84) | 0.53 (0.17 to 1.18) |
| Excluding TKR or UKR studies | 12 | 23.68 (16.36 to 33.17) | 0.53 (0.17 to 1.29) |
| Excluding studies with > 20% loss to follow-up | 11 | 26.13 (18.08 to 36.46) | 0.49 (0.14 to 1.25) |
| Excluding studies with overall high risk of bias | 12 | 25.01 (17.87 to 34.74) | 0.48 (0.16 to 1.17) |
| 6 Months | | | |
| Excluding Mekkawy 2023 and Yan 2023 | 26 | 13.97 (10.74 to 18.13) | 0.54 (0.27 to 0.95) |
| Excluding TKR or UKR studies | 26 | 14.24 (10.88 to 18.46) | 0.54 (0.27 to 0.95) |
| Excluding studies with > 20% loss to follow-up | 19 | 16.78 (12.37 to 22.52) | 0.52 (0.22 to 1.03) |
| Excluding studies with overall high risk of bias | 19 | 15.63 (11.25 to 21.19) | 0.58 (0.24 to 1.12) |
| 12 Months | | | |
| Excluding Fast track studies | 34 | 12.15 (9.5 to 15.15) | 0.55 (0.3 to 0.91) |
| Excluding TKR or UKR studies | 35 | 12.72 (9.85 to 16) | 0.63 (0.35 to 1.01) |
| Excluding studies with > 20% loss to follow-up | 19 | 15.3 (11.09 to 21.01) | 0.58 (0.23 to 1.16) |
| Excluding studies with overall high risk of bias | 20 | 14.37 (10.14 to 19.49) | 0.65 (0.28 to 1.23) |
| 24 Months | | | |
| Excluding Leppanen 2021 | 9 | 13.78 (8.33 to 21.28) | 0.52 (0.15 to 1.45) |
| Excluding TKR or UKR studies | 9 | 13.18 (8.59 to 20.26) | 0.42 (0.11 to 1.18) |
| Excluding studies with > 20% loss to follow-up | 6 | 18.74 (9.79 to 33.5) | 0.59 (0.11 to 2.29) |
| Excluding studies with overall high risk of bias | 7 | 15.28 (8.68 to 26.24) | 0.53 (0.12 to 1.78) |

*Abbreviation: TKR: Total Knee Replacement; UKR: Unicompartmental Knee Replacement

# S11. Characteristics of THR studies

| **Study**  **Country**  **Recruitment dates**  **Setting** | **Operation**  **Number of patients**  **Age (SD), range**  **% women** | **Pain measure** | **Definition of unfavourable pain outcome**  **High risk of bias concern** |
| --- | --- | --- | --- |
| Cleveland Clinic OME Arthroplasty Group 2020[1]  USA  2015-2018  6 hospitals | Primary THR, all  N=3449  Median 65 (IQR 57-72)  57.4% | HOOS pain  12 months | Less than MCID (15 points) |
| Erlenwein 2017[2]  Germany  2012  1 hospital | Primary THR, all 18+  N=125  63 (12.6)  58% | NRS pain  6 months | Maximum NRS >3 during previous 4 weeks |
| Jones 2000[3]  Canada  1995-1997  1 health region | Primary THR, all 40+  N=242  68.2 (11.1)  60% | WOMAC pain  6 months | Moderate/ severe pain defined as a gain of <10 points on the WOMAC pain dimension |
| Mezey 2023[4]  Hungary  2019-2020  2 hospitals | Primary THR, all  N=88  68.7 (THR and TKR patients)  69.2% | WOMAC pain  12 months | Not exceeding MCID (8.3)  High loss to follow up rate |
| Nikolajsen 2006[5]  Denmark  2003  National registry | Primary THR, 18-90 years  N=1231  71.6 (8.7)  Not reported | Authors’ own scale of presence of hip pain and impact on daily life  12-18 months | Pain with moderate, severe or very severe impact on daily life |
| Page 2016[6]  Canada  2009-2012  1 hospital | Primary THR, all 18-75  N=150  60 (9.2)  48% | Authors’ own scale  6 months | Chronic pain if pain rated as “discomforting”, “distressing”, “horrible,” or “excruciating”  Concern as RCT analysed as cohort study |
| Palazzo 2014[7]  France  2009  3 hospitals | Primary THR, all  N=129  63.5 (13.5)  49.6% | Author’s own residual pain scale  12 months | “To what extent have you obtained a relief or improvement as a result of THA in the following areas?” (from 0: not at all; to 4: completely) |
| Quintana 2006[8]  Spain  1999-2000  7 hospitals | Primary THR  N=784  69.1  48.3% | WOMAC pain  6 months | No improvement in pain greater than MCID (24.55 of 100) using an anchor-based method.  Concern for high loss to follow up rate |
| Ray 2020[9]  Sweden  2008-2015  National registry | THR  N= 127,660  68 (10)  56% | EQ-5D VAS pain/discomfort  12 months | Worse or no change in pain/discomfort  Concern for high loss to follow up rate |
| Singh and Lewallen 2010[10]  USA  1993-2005 | Primary THR  N=9154  65 (13.3)  51% | Authors’ own scale: How much pain do you have in your operated hip? None, mild, moderate or severe  24 months | Moderate or severe pain  Concerns for high loss to follow up rate |
| Tang 2023[11]  China  2020-2021  1 hospital | Primary THR probably, all 65+. Osteoarthritis or osteonecrosis (not fracture)  N=89  72 (range 63-81)  62.5% | NRS pain  3 months | NRS scores ≥4  Note, n and losses to follow up estimated as proportions because n hips and knees reported together |

## References

1. Cleveland Clinic OME Arthroplasty Group, Arnold N, Anis H, Barsoum WK, Bloomfield MR, Brooks PJ, Higuera CA, Kamath AF, Klika A, Krebs VE, Mesko NW, Molloy RM, Mont MA, Murray TG, Patel PD, Strnad G, Stearns KL, Warren J, Zajichek A, Piuzzi NS. Preoperative cut-off values for body mass index deny patients clinically significant improvements in patient-reported outcomes after total hip arthroplasty. Bone Joint J. 2020;102-B(6):683-92.

2. Erlenwein J, Muller M, Falla D, Przemeck M, Pfingsten M, Budde S, Quintel M, Petzke F. Clinical relevance of persistent postoperative pain after total hip replacement - a prospective observational cohort study. J Pain Res. 2017;10:2183-93.

3. Jones CA, Voaklander DC, Johnston DW, Suarez-Almazor ME. Health related quality of life outcomes after total hip and knee arthroplasties in a community based population. J Rheumatol. 2000;27(7):1745-52.

4. Mezey GA, Paulik E, Mate Z. Effect of osteoarthritis and its surgical treatment on patients' quality of life: a longitudinal study. BMC Musculoskelet Disord. 2023;24(1):537.

5. Nikolajsen L, Brandsborg B, Lucht U, Jensen TS, Kehlet H. Chronic pain following total hip arthroplasty: a nationwide questionnaire study. Acta Anaesthesiol Scand. 2006;50(4):495-500.

6. Page MG, Katz J, Curtis K, Lutzky-Cohen N, Escobar EM, Clarke HA. Acute pain trajectories and the persistence of post-surgical pain: a longitudinal study after total hip arthroplasty. J Anesth. 2016;30(4):568-77.

7. Palazzo C, Jourdan C, Descamps S, Nizard R, Hamadouche M, Anract P, Boisgard S, Galvin M, Ravaud P, Poiraudeau S. Determinants of satisfaction 1 year after total hip arthroplasty: the role of expectations fulfilment. BMC Musculoskelet Disord. 2014;15:53.

8. Quintana JM, Escobar A, Arostegui I, Bilbao A, Azkarate J, Goenaga JI, Arenaza JC. Health-related quality of life and appropriateness of knee or hip joint replacement. Arch Intern Med. 2006;166(2):220-6.

9. Ray GS, Ekelund P, Nemes S, Rolfson O, Mohaddes M. Changes in health-related quality of life are associated with patient satisfaction following total hip replacement: an analysis of 69,083 patients in the Swedish Hip Arthroplasty Register. Acta Orthop. 2020;91(1):48-52.

10. Singh JA, Lewallen D. Predictors of pain and use of pain medications following primary Total Hip Arthroplasty (THA): 5,707 THAs at 2-years and 3,289 THAs at 5-years. BMC Musculoskelet Disord. 2010;11:90.

11. Tang S, Jin Y, Hou Y, Wang W, Zhang J, Zhu W, Zhang W, Gu X, Ma Z. Predictors of Chronic Pain in Elderly Patients Undergoing Total Knee and Hip Arthroplasty: A Prospective Observational Study. J Arthroplasty. 2023;38(9):1693-9.

## S11.1 Proportions of lost to follow-ups and revisions


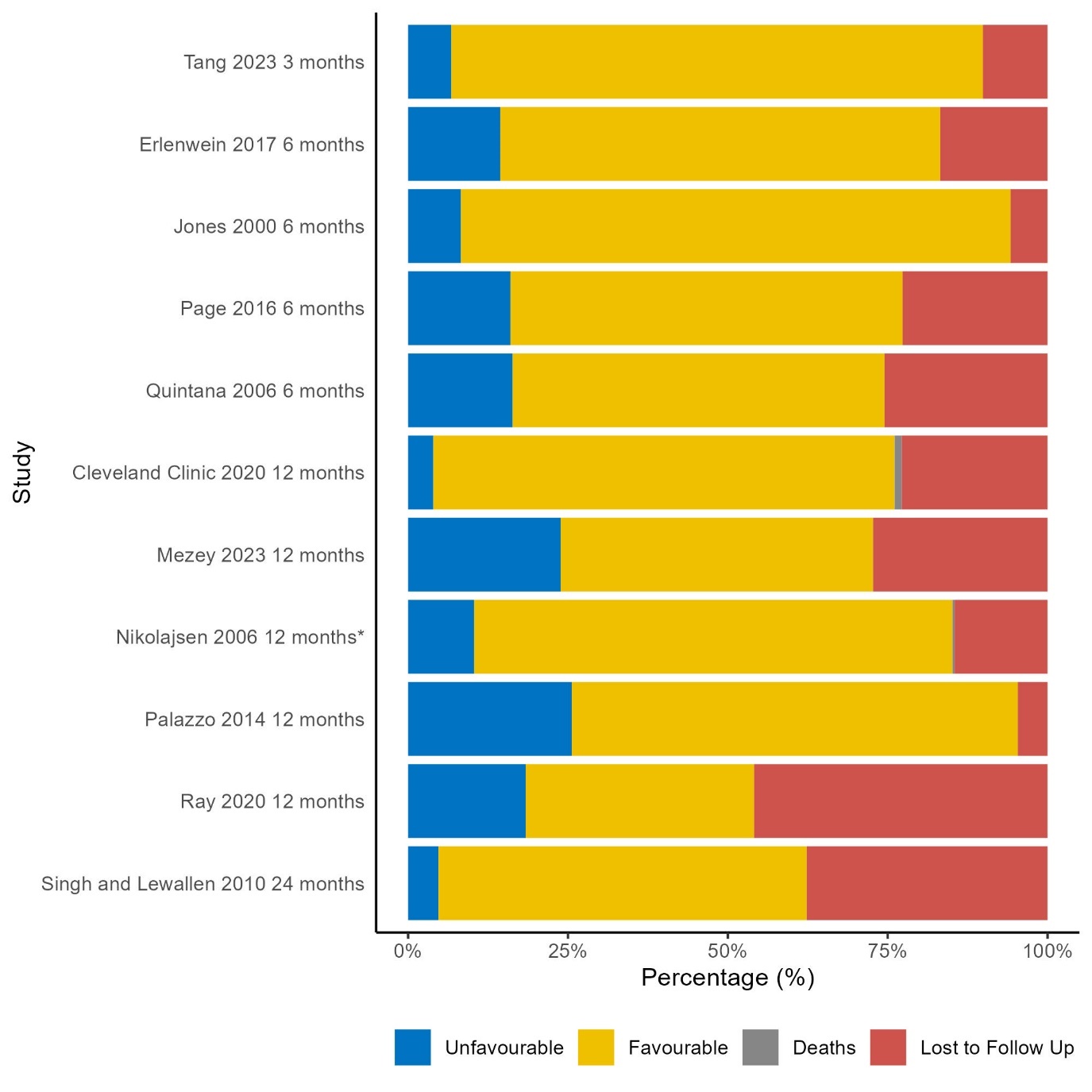


Figure S12.1. Favourable and unfavourable pain outcomes and reasons of missing data in THR studies.

# S12. Traffic light plot of the risk of bias assessments in THR studies


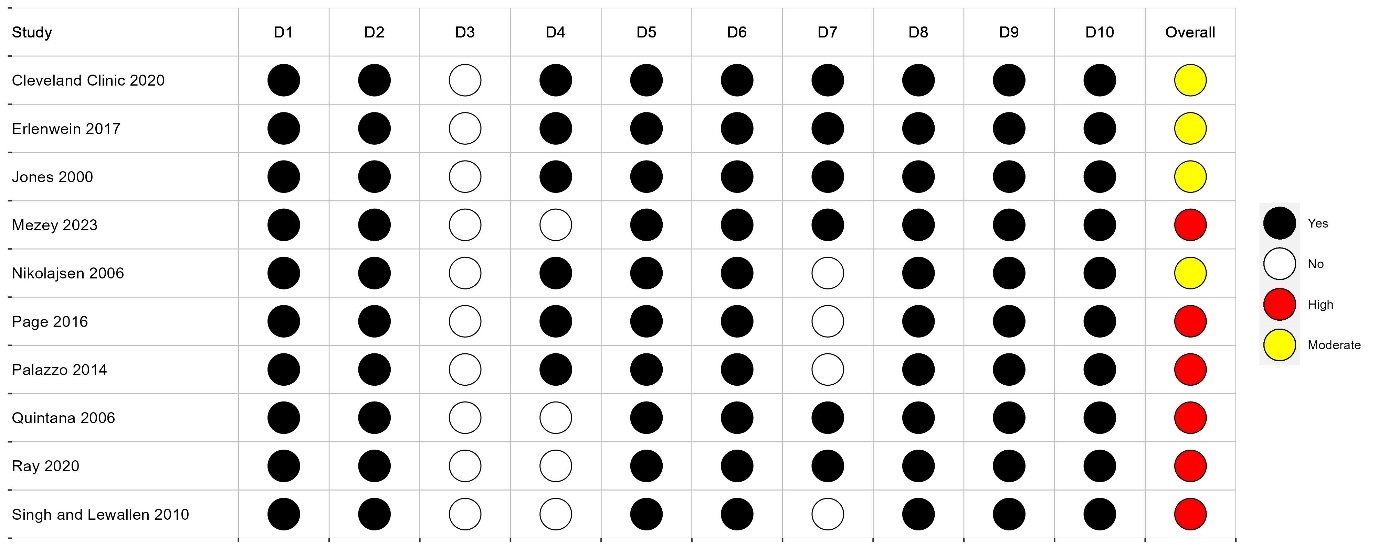

Supplement: online supplemental file 1 [file bmjopen-15-5-s001.docx]
