# Supplementary material for: What proportion of people have long-term pain after total hip or knee replacement? An update of a systematic review and meta-analysis
Source: BMJ Open. 2025 May 21;15(5):e088975. doi: 10.1136/bmjopen-2024-088975 (PMC12096998; doi:10.1136/bmjopen-2024-088975)
Supplement: Supplementary file 2 [file bmjopen-2024-088975.reviewer_comments.pdf]

PEER REVIEW HISTORY

BMJ Open publishes all reviews undertaken for accepted manuscripts. Reviewers are asked to complete a checklist review form and are provided with free text boxes to elaborate on their assessment. These free text comments are reproduced below.

ARTICLE DETAILS

Title (Provisional)

What proportion of people have long-term pain after total hip or knee replacement? An update of a systematic review and meta-analysis

Authors

Cheng, Hung-Yuan; Beswick, Andrew David; Bertram, Wendy; Siddiqui, Mohammad Ammar; Gooberman-Hill, Rachael; Whitehouse, Michael R; Wylde, Vikki

---

VERSION 1 - REVIEW

**Reviewer**	**1**
**Name**	**Katz, Jeffrey**
**Affiliation**	**Brigham and Women's Hospital, Orthopaedic and Arthritis Center for Outcomes Research**
**Date**	**09-Aug-2024**
**COI**	**None**

The authors follow up their prior review of residual pain following TKR and THR by updating it with papers published after 2011. The methods are rigorous and well described, in general, but the primary outcome is problematic as currently presented.

The authors indicate in Table 1 which outcome measures were used in the reviewed studies. e.g. VAS and WOMAC Pain. In the appendix (S3) hey indicate how the binary 'unfavorable outcome' was defined. The analyses and conclusions are based on the binary 'unfavorable outcomes.'

The definitions of unfavorable used in each study (S.3) vary widely and in some cases are not provided with sufficient specificity. E.g. where the authors indicate "MCID not satisfied" they should state the MCID used. Same with PASS.

The authors should examine the relationship between how the binary variable is defined and the proportion with 'unfavorable outcomes.'

Please see Musbahi and colleagues on this topic (10.2106/JBJS.OA.23.00077; JBJS Open Access, 2023). These authors (I am an author) used data from a high quality prospective cohort and defined residual pain at 12 months after TKR using a range of metrics (eg various

cutoffs for PASS, MCID, WOMAC Pain thresholds and others). The frequency of unfavorable pain outcomes varied from 5% to 50% with these various definitions. The proportion dissatisfied with TKR was just 6% in the cohort. So most subjects with 'unfavorable' pain outcomes were satisfied with surgery.

Your paper should address this issue. I might suggest that you group together sturdies using more stringent and those using less stringent definitions of unfavorable pain outcome and examine whether (as might be expected) the frequency of unfavorable pain reflects the stringency of the outcome.

Many readers have interpreted your work to say that unfavorable pain outcome equates with failure. The satisfaction data indicate that is not the case. i think your analyses and discussion should address this issue clearly.

**Reviewer**	**2**
**Name**	**de Steiger, Richard**
**Affiliation**	**Epworth HealthCare, Department of Surgery**
**Date**	**26-Aug-2024**
**COI**	**NONE**

This is a well written systematic review, and I only have minor comments.

METHODS

Outcome

I am a little unclear by ‘unfavourable pain’. It is defined in the introduction as moderate to severe where surgery had not relieved pain. I presume that this refers to the same joint that was operated on and not a general VAS pain score that may be related to other joints, back pain etc. I assume this was clearly stated in the papers in the synthesis.

RESULTS

Figure 1: It is only for this reviewer’s interest for such an extensive review and considering the researchers’ track record, I was interested to know why one publication in the TKR section was unobtainable. Was it an obscure publication, language other than English that couldn’t be translated by Google? The reviewers don’t have to answer but I was curious.

Table 1: I am surprised only 2 National registry studies included. I would have thought Scandinavian countries would have contributed more to this topic. Maybe the studies form part of multiple hospital studies rather than Registry based.

DISCUSSION

The authors have clearly outlined limitations and discussed in detail the evidence around prevention of chronic pain. While this is not directly related to the research question, it does provide a valuable overview and fits the aims of this thorough review.

It is well documented that poorer PROMs (with the pain subset in particular) is a predictor of a higher revision rate. While these studies excluded patients with revisions, it is probably worth commenting on this as patients with chronic pain especially at 6-12 months, may not have undergone revision but may have at a later time period.

Also, may be worth commenting that, despite the prevalence of unfavourable pain at delayed time points TKR 'works' (RCT of TKR - SKOU ST et al NEJM 2015 373). Should it be the change in pain from pre to post operative that we should be measuring rather than the patient reported pain at a particular time point.

VERSION 1 - AUTHOR RESPONSE

2. Reviewer: 1

Prof. Jeffrey Katz, Brigham and Women's Hospital

2.1 Reviewer comment

Comments to the Author:

The authors follow up their prior review of residual pain following TKR and THR by updating it with papers published after 2011. The methods are rigorous and well described, in general, but the primary outcome is problematic as currently presented.

The authors indicate in Table 1 which outcome measures were used in the reviewed studies. e.g. VAS and WOMAC Pain. In the appendix (S3) they indicate how the binary 'unfavorable outcome' was defined. The analyses and conclusions are based on the binary 'unfavorable outcomes.'

The definitions of unfavorable used in each study (S.3) vary widely and in some cases are not provided with sufficient specificity. E.g. where the authors indicate "MCID not satisfied" they should state the MCID used. Same with PASS.

2.1 Response: Thank you for raising this issue, which we agree is of particular importance for the current study. We have revised the Supplementary materials (S3 and S11) to include definitions of the MCID and PASS used in the included studies.

2.2 Reviewer comment

The authors should examine the relationship between how the binary variable is defined and the proportion with 'unfavorable outcomes.'

2.2 Response: We acknowledge the importance of understanding the relationship between how the binary variable is defined and the proportion of patients with "unfavourable pain" in the context of this review. However, unlike Musbahi et al., we do not have access to individual patient data, which limits our ability to investigate the relationship between pain and satisfaction. Additionally, very few of the included studies provide multiple cut-off points for such an examination.

We have addressed this further in response to comment 2.4.

2.3 Reviewer comment

Please see Musbahi and colleagues on this topic (10.2106/JBJS.OA.23.00077; JBJS Open Access, 2023). These authors (I am an author) used data from a high quality prospective cohort and defined residual pain at 12 months after TKR using a range of metrics (e.g. various cutoffs for PASS, MCID, WOMAC Pain thresholds and others). The frequency of unfavorable pain outcomes varied from 5% to 50% with these various definitions. The proportion dissatisfied with TKR was just 6% in the cohort. So most subjects with 'unfavorable' pain outcomes were satisfied with surgery.

2.3 Response: Thank you for highlighting this point. We agree with you that pain and dissatisfaction are two different constructs as discussed in a systematic review (Klem et al., 2020; doi: 10.1016/j.ocarto.2020.100032) and other qualitative studies (Klem et al., 2021; doi: 10.1136/bmjopen-2021-050385; Klem et al., 2020; doi: 10.1097/CORR.0000000000001284; and Woolhead et al., 2005; doi: 10.1093/rheumatology/keh674).

We adopted the term "unfavourable pain" from the previous review. The terminology had been piloted by authors Beswick and Dieppe of the 2012 article at conferences in the UK and US (ACR/ARHP 2011; available from: https://acrabstracts.org/wp-content/uploads/2018/06/2011_ACR_ARHP_Abstract_Supplement.pdf). It serves as a collective term to include the various descriptions used by study authors, such as persistent pain, unchanged pain, worsening pain or residual pain, rather than as an indicator of dissatisfaction. A large majority of studies provide a single estimate of "unfavourable pain" (see 2.4 Response below). For a few studies, we found it necessary to select from a series of cut-offs based on study results and conclusions. For example, in the study of Musbahi and colleagues, we chose the WOMAC pain score improvement of <20.0 which the authors concluded "*appeared to provide the best balance between sensitivity and specificity with respect to predicting participant dissatisfaction, with a Youden index of 49%*".

2.4 Reviewer comment

Your paper should address this issue. I might suggest that you group together studies using more stringent and those using less stringent definitions of unfavorable pain outcome and examine whether (as might be expected) the frequency of unfavorable pain reflects the stringency of the outcome.

Many readers have interpreted your work to say that unfavorable pain outcome equates with failure. The satisfaction data indicate that is not the case. I think your analyses and discussion should address this issue clearly.

2.4 Response: Thank you for these points and suggestions above. We have reflected on these points and amended the manuscript as follows:

a. Definition of our primary outcome, 'unfavourable pain': we have amended Introduction (Lines 89-94; 97-98, Page 4) to explain that pain and dissatisfaction are two distinct constructs. We have also clarified our outcome definitions in Methods (Lines 129-133; 136-140, Page 5) as mentioned in 2.3 Response. We have also acknowledged this as a limitation of the review (Lines 372-376, Page 13).

b. Grouping studies based on stringent definitions of outcomes: we agree that variations in outcome definitions contribute to heterogeneity in the current review as you raised. In addition to the subgroup analysis on the type of pain outcome instruments in TKR studies (S8.3), we have conducted an additional analysis to examine the definitions of cut-offs in the included studies. We categorised the cut-off definitions as "based on MCID", "based on PASS", "based on symptom improvement", "based on functional impact", and "based on pain intensity" (S8.4). The distribution of different cut-off definitions is skewed, often relying on less stringent definitions, such as a single post-operative VAS/NRS score, rather than more robust measures, like MCID or PASS.

We amended the results section (Line 271-291, Page 10) to include the results of the subgroup analysis and amended the discussion accordingly (Line 341-354, Pages 12-13). In the discussion, we now acknowledge the value of primary analyses of cohort studies as reported in Musbahi 2023, and other exploratory studies of patient experiences. A description of the range of WOMAC pain thresholds using MCID and PASS described in Musbahi 2023 is also presented. There are not enough THR studies (only 11 studies in total) to provide meaningful subgroup analysis.

3. Reviewer: 2

Dr. Richard de Steiger, Epworth HealthCare

3.1 Reviewer comment

Comments to the Author:

This is a well written systematic review, and I only have minor comments.

METHODS

Outcome

I am a little unclear by 'unfavourable pain'. It is defined in the introduction as moderate to severe where surgery had not relieved pain. I presume that this refers to the same joint that was operated on and not a general VAS pain score that may be related to other joints, back pain etc. I assume this was clearly stated in the papers in the synthesis.

3.1 Response: Thank you for asking about how we define unfavourable pain. The use of this term in our previous review was to differentiate from people who had a favourable pain outcome. We have now noted this in the introduction section and hope this makes the concept of 'unfavourable pain' clearer.

In revisions to the manuscript we have edited the text and added a clear definition (Lines 140-142, Page 5) as well as highlighting the diversity of approaches in the included studies (Lines 130-133, Page 5).

3.2 Reviewer comment

RESULTS

Figure 1: It is only for this reviewer's interest for such an extensive review and considering the researchers' track record, I was interested to know why one publication in the TKR section was unobtainable. Was it an obscure publication, language other than English that couldn't be translated by Google? The reviewers don't have to answer but I was curious.

3.2 Response: Thank you, this is an interesting question. We could not access the full text of one publication and exhausted multiple resources in our attempts to do so, including through librarians and researchers in the team. From the abstract of that article, we could not ascertain how the cohort of 72 patients was selected, although all had completed a rehabilitation programme.

3.3 Reviewer comment

Table 1: I am surprised only 2 National registry studies included. I would have thought Scandinavian countries would have contributed more to this topic. Maybe the studies form part of multiple hospital studies rather than Registry based.

3.3 Response: Thank you for the comment. Many national registry studies did not provide dichotomous results or percentages to estimate the proportion of participants

with unfavourable pain, which meant that their results could not be included in our review. We have now addressed this in the Discussion and noted that "*Registry studies are a potentially rich and largely untapped source of data from representative populations for the exploration of patient and healthcare factors in relation to chronic pain*" (Lines 445-447, Page 15).

3.4 Reviewer comment

DISCUSSION

The authors have clearly outlined limitations and discussed in detail the evidence around prevention of chronic pain. While this is not directly related to the research question, it does provide a valuable overview and fits the aims of this thorough review.

It is well documented that poorer PROMs (with the pain subset in particular) is a predictor of a higher revision rate. While these studies excluded patients with revisions, it is probably worth commenting on this as patients with chronic pain especially at 6-12 months, may not have undergone revision but may have at a later time period.

3.4 Response: Thank you for asking about the exclusion of patients with revisions. To make this clearer, we have amended the discussion to include information that patients with chronic pain are likely to undergo revision at a later time period (Lines 403-404, Page 14). We have also noted that "*at 12 months after TKR when patients should have recovered from surgery and be largely unaffected by issues relating to implant failure…*" (Lines 322-324, Page 12) in Discussion.

3.5 Reviewer comment

Also, may be worth commenting that, despite the prevalence of unfavourable pain at delayed time points TKR 'works' (RCT of TKR - SKOU ST et al NEJM 2015 373). Should it be the change in pain from pre to post operative that we should be measuring rather than the patient reported pain at a particular time point.

3.5 Response: Thank you. As also noted by Reviewer 1, the included studies used a variety of cut-offs to dichotomise unfavourable pain, including improvement in pain symptoms from pre-operative to post-operative. Our review therefore reflects the diversity of approaches in the literature. We have made revisions to the manuscript to clarify this (see 2.4 Response).

VERSION 2 - REVIEW

**Reviewer**	**2**

**Name**	**de Steiger, Richard**
**Affiliation**	**Epworth HealthCare, Department of Surgery**
**Date**	**06-Dec-2024**
**COI**	

I am happy with the author's response to my review and for the changes made to the manuscript.

Unfortunately, as often happens, the responses do not match the pages/lines in the manuscript, but all OK.

**Reviewer**	**3**
**Name**	**Martin, James**
**Affiliation**	**University of Birmingham, Institute of Applied Health Research**
**Date**	**14-Mar-2025**
**COI**	**None**

Thank you for submitting this revised manuscript. I have only a few minor comments.

In the conclusions section of the abstract, you refer to the 22% of patients reporting unfavourable pain 3 months post-TKR. From the abstract, it's not clear what "unfavourable pain" is, and whether this is the same, or different from "long-term pain". Otherwise at this point, a reader may be confused as I would think that all pain is "unfavourable".

On line 104, you refer to it being twelve years since the publication of the previous review. I think that was published in 2012, so it should now be thirteen years since that review.

For the exclusion reasons listed for studies related to THR, you list "not consecutive" as a reason for exclusion. I just wondered if you could clarify what you mean by this.

The range of people with unfavourable pain across studies at each time-point varies widely. Do you think this is solely due to differing definitions of unfavourable pain outcomes?

VERSION 2 - AUTHOR RESPONSE

2. Reviewer: 2

Prof. Richard de Steiger, Epworth HealthCare

Comments to the Author:

I am happy with the author's response to my review and for the changes made to the manuscript.

Unfortunately, as often happens, the responses do not match the pages/lines in the manuscript, but all OK.

2 Response: Thank you for your positive feedback and for reviewing our manuscript again. We did our best to align responses with the latest version of the manuscript to address this concern.

3. Reviewer: 3

Dr. James Martin, University of Birmingham

Comments to the Author:

Thank you for submitting this revised manuscript. I have only a few minor comments.

3.1 In the conclusions section of the abstract, you refer to the 22% of patients reporting unfavourable pain 3 months post-TKR. From the abstract, it's not clear what "unfavourable pain" is, and whether this is the same, or different from "long-term pain". Otherwise at this point, a reader may be confused as I would think that all pain is "unfavourable".

3.1 Response: Apologies for this. We have removed "unfavourable".

3.2 On line 104, you refer to it being twelve years since the publication of the previous review. I think that was published in 2012, so it should now be thirteen years since that review.

3.2 Response: Thank you, this has been changed to thirteen.

3.3 For the exclusion reasons listed for studies related to THR, you list "not consecutive" as a reason for exclusion. I just wondered if you could clarify what you mean by this.

3.3 Response: To ensure that populations included in our review were representative of all hip or knee replacements in each centre, we screened out studies where only a proportion of patients were included. In the case of this THR study, a cohort of 72 patients were included between 2002 and 2008. They were assessed before surgery and also at 12 months. There was no information on patients not followed up and thus patients were not consecutive. In fact, this study could also have been excluded as a "Selected group".

3.4 The range of people with unfavourable pain across studies at each time-point varies widely. Do you think this is solely due to differing definitions of unfavourable pain outcomes?

3.4 Response: We were unable to determine whether the variation in the proportion of people with unfavourable pain across studies at each time-point is solely due to differing definitions of unfavourable pain outcomes. In our subgroup analysis (see Supplementary S8.3), we attempted to group studies by the type of pain outcome instrument used to explore this issue. However, the number of studies within each group, whether using similar or different instruments, was too small to draw any reliable conclusions. Similarly, inspection of the individual meta-analysis plots (arranged by pain outcome instrument; see Supplementary Figures S5.1–S5.4) did not reveal any clear contribution of the pain measurement tool to the observed heterogeneity.
